# Bayesian model averaging for partial ordering continual reassessment methods

**DOI:** 10.1093/biostatistics/kxaf035

**Published:** 2025-10-21

**Authors:** Luka Kovačević, Weishi Chen, Helen Barnett, Thomas Jaki, Pavel Mozgunov

**Affiliations:** MRC Biostatistics Unit, East Forvie Building, Forvie Site, Robinson Way, Cambridge Biomedical Campus, Cambridge CB2 0SR, United Kingdom; MRC Biostatistics Unit, East Forvie Building, Forvie Site, Robinson Way, Cambridge Biomedical Campus, Cambridge CB2 0SR, United Kingdom; School of Mathematical Sciences, Fylde College, Lancaster University, Lancaster, LA1 4YF, United Kingdom; MRC Biostatistics Unit, East Forvie Building, Forvie Site, Robinson Way, Cambridge Biomedical Campus, Cambridge CB2 0SR, United Kingdom; Department of Machine Learning and Data Science, University of Regensburg, Bajuwarenstrasse 4, Regensburg, 93053, Germany; MRC Biostatistics Unit, East Forvie Building, Forvie Site, Robinson Way, Cambridge Biomedical Campus, Cambridge CB2 0SR, United Kingdom

**Keywords:** adaptive design, Bayesian inference, incoherence, maximum tolerable dose

## Abstract

Phase I clinical trials are essential to bringing novel therapies from chemical development to widespread use. Traditional approaches to dose-finding in Phase I trials, such as the ‘3 + 3’ method and the continual reassessment method (CRM), provide a principled approach for escalating across dose levels. However, these methods lack the ability to incorporate uncertainty regarding the dose-toxicity ordering as found in combination drug trials. Under this setting, dose levels vary across multiple drugs simultaneously, leading to multiple possible dose-toxicity orderings. The CRM for partial ordering (POCRM) extends to these settings by allowing for multiple dose-toxicity orderings. In this work, it is shown that the POCRM is vulnerable to ‘estimation incoherency’ whereby toxicity estimates shift in an illogical way, threatening patient safety and undermining clinician trust in dose-finding models. To this end, the Bayesian model averaged POCRM (BMA-POCRM) is formalized. BMA-POCRM uses Bayesian model averaging to take into account all possible orderings simultaneously, reducing the frequency of estimation incoherencies. We derive novel theoretical guarantees on the estimation coherency of the POCRM and BMA-POCRM. The effectiveness of BMA-POCRM in drug combination settings is demonstrated through a specific instance of estimate incoherency of POCRM and simulation studies. The results highlight the improved safety, accuracy, and reduced occurrence of estimate incoherency in trials applying the BMA-POCRM relative to the POCRM model.

## 1. INTRODUCTION

An aim of in-patient Phase I clinical trials is to determine the maximum tolerable dose (MTD) from a range of dose levels for progression into Phase II trials. The MTD is the dose level that matches the target probability of unwanted effects, sometimes called the target toxicity rate (TTR). Depending on the setting, the MTD can be either a dose level for a single agent or a combination of dose levels in a multi-agent setting. A common approach to determining the MTD is to use the number of dose-limiting toxicities (DLTs), simply called toxicities, on each dose to compute a probability of toxicity. The definition of a DLT and the desired TTR are set in advance of a trial, hence, by estimating the risk of DLT for each dose, the dose level with a toxicity rate closest to the TTR can be selected. Dose-escalation procedures aim to estimate the location of the true MTD given a TTR and sequential patient DLT data.

Approaches to dose-escalation in Phase I clinical trials frequently rely on the assumption of simple dose orderings. In the case of single drug Phase I trials, a simple ordering can be constructed by assuming a monotonic increasing relationship between dose level and toxicity. That is, higher doses of a drug are assumed to be more toxic than lower doses. For example, suppose there are dose levels $ d_{1},\ldots, d_{4} $, where a higher index indicates a higher dose level. This means that $ d_{1} \lt\ldots < d_{4} $ implies the following simple ordering assuming dose-toxicity monotonicity,


\begin{align*} d_{1}\rightarrow d_{2}\rightarrow d_{3}\rightarrow d_{4}.\end{align*}

This also implies that $ d_{2} $ is more toxic than $ d_{1} $, $ d_{3} $ is more toxic than both $ d_{1} $ and $ d_{2} $ and so forth. The continual reassessment method (CRM) is one such approach that is based on the assumption of monotonicity. It utilizes a Bayesian framework to update estimates of the risk of toxicity to guide dose-escalation based on a given toxicity ordering of doses (O’Quigley et al. 1990). Several independent studies have shown that escalation based on the CRM leads to favourable operating characteristics for finding the true MTD in single-agent clinical trials. With the growing need for combination drug trials, where the toxicity profile of joint administration of 2 or more drugs is investigated, methods that allow for potential uncertainties in the ordering of dose levels are necessary (Mozgunov et al. 2020).

Escalation of multiple drugs concurrently creates uncertainty in the dose-escalation process as the change in dose toxicity is not obvious for diagonal transitions in dose level, eg where one drug increases in dose level and the other decreases. Specifically, it may be reasonable to assume monotonicity for a single drug, however, this does not extend to multiple changes in dose level. In Table 1, the 3-by-2 dose level configuration for a dual drug combination trial with 3 dose levels for drug $ A $ and 2 dose levels for drug $ B $ is shown. Here, it is unknown prior to the trial whether toxicity increases or decreases with a shift between $ d_{2} $ and $ d_{3} $ and likewise for $ d_{4} $ and $ d_{5} $. Based on this dose-level matrix, the 5 simple orderings comprising the partial ordering of dose levels are,


\begin{align*} 1 : \; d_{1}\rightarrow d_{2}\rightarrow d_{3}\rightarrow d_{4}\rightarrow d_{5}\rightarrow d_{6},\\2 : \; d_{1}\rightarrow d_{3}\rightarrow d_{5}\rightarrow d_{2}\rightarrow d_{4}\rightarrow d_{6},\\3 : \; d_{1}\rightarrow d_{3}\rightarrow d_{2}\rightarrow d_{5}\rightarrow d_{4}\rightarrow d_{6},\\4 : \; d_{1}\rightarrow d_{2}\rightarrow d_{3}\rightarrow d_{5}\rightarrow d_{4}\rightarrow d_{6},\\5 : \; d_{1}\rightarrow d_{3}\rightarrow d_{2}\rightarrow d_{4}\rightarrow d_{5}\rightarrow d_{6}.\end{align*}

**Table 1.** $ 3\times 2 $ setting resulting in 6 dose levels.

		Drug B	
		1	2
Drug A	3	$ \boldsymbol{d}_{\bf 5} $	$ \boldsymbol{d}_{\bf 6} $
	2	$ \boldsymbol{d}_{\bf 3} $	$ \boldsymbol{d}_{\bf 4} $
	1	$ \boldsymbol{d}_{\bf 1} $	$ \boldsymbol{d}_{\bf 2} $

Several procedures have been developed to handle the problem of uncertain dose-toxicity orderings, which is a problem that also persists in dose-schedule (Mozgunov and Jaki 2019) and combination-schedule settings (Riviere et al. 2015; Mozgunov et al. 2022). Ivanova and Wang (2004) developed the *up-and-down design for combinations* using an isotonic regression combined with the Narayana design to escalate doses algorithmically (Ivanova et al. 2003). A further development updated this method to utilize a *T-statistic* (Ivanova and Kim 2009) for escalation decisions. Furthermore, Yin and Yuan (2009) have developed the Bayesian copula regression-based model, which uses a Bayesian scheme similar to the CRM to update posterior estimates of toxicity and recommend dose allocation decisions. Most recently, Mozgunov et al. (2020) proposed a beta-distributed surface-free approach to handling drug combination trials, successful in reducing the average number of toxicities within a trial. The CRM for partial orderings (POCRM), developed by Wages et al. (2011), extends the Bayesian framework of the CRM to allow for several dose-toxicity orderings to be specified. For a more complete reporting of recently developed adaptive methods for Phase I clinical trials, we refer the reader to Barnett et al. (2024) and Liu et al. (2024).

Furthermore, the POCRM addresses the problem of uncertainty in dose-toxicity orderings by selecting from the set of proposed dose-toxicity orderings for each cohort in the trial. Given a set of simple orderings that are pre-specified, POCRM selects the most likely ordering and uses this to recommend the next dose to be assigned in the trial via the CRM. This approach is particularly favourable as it allows for flexibility in the dose-toxicity ordering used for dose recommendation. However, it also significantly limits the performance of the model as, particularly in cases with a large number of dose combinations, not all possible orderings can be considered by the model since the model can only consider orderings given a priori. It can be argued that the true MTD will be selected regardless of whether the correct dose ordering is present, however, since the proposed orderings alter toxicity estimates, they also guide dose-escalation. In the combination setting, multiple doses that have a toxicity close to the TTR may be present. Therefore, inaccurate estimation of the risk of toxicity may lead to the incorrect conclusion that there is only a single potential MTD. In this case, not only is escalation important but also point estimation of dose toxicity rates. Another instance under which this method can pose challenges is where multiple orderings have a similar posterior probability leading to the uncertainty in dose orderings being disregarded as only the model with the highest posterior probability is selected at each step. This article also explores the prevalence of illogical large changes in dose-toxicity estimates present in practice caused by shifts in the simple ordering with the greatest posterior probability.

To address the challenges associated with ordering selection and uncertainty quantification during trials, the original POCRM is extended by applying Bayesian model averaging (BMA) in this work. Previously, BMA has been applied to the original CRM (Yin and Yuan 2009), where it was highly successful in improving dose allocation for single-agent trials, particularly for small sample Phase I trials (Conaway et al. 2004). The novel Bayesian model averaged POCRM (BMA-POCRM) design, that we propose in this work, aims to incorporate uncertainty in the toxicity ordering with the aim of making more flexible dose-toxicity estimates, which are not limited to following a single predefined ordering, and take into account the additional uncertainty implied by a partial ordering.

Previous work by Zhang et al. (2023) has considered the application of BMA to the POCRM for a different problem to the one considered here. They consider the problem of a large number of combination levels, resulting in the original POCRM being hard to specify or having poor operating characteristics due to a small spacing between skeleton values. To tackle this, the authors apply CRM only to a subset of combination levels and then use model averaging within that ‘local’ space. Instead, in this article, we consider averaging across the whole grid that concerns the original POCRM. Another distinction of this work to Zhang et al. (2023) is that we propose to apply BMA to the ordering-specific posterior distributions of the toxicity probabilities to obtain an averaged (mixture) posterior distribution of toxicity probabilities. This is opposed to applying BMA to the ordering-specific point estimates of the toxicity probabilities proposed in Zhang et al. (2023). We argue that it is beneficial to work with the mixture posterior as it can be directly used to derive other summary quantities, such as 95% credible interval around the point estimates, and the probability of overdosing. Another approach for the BMA was proposed by Wages et al. (2011), which apply BMA to the ordering-specific recommended doses and is fundamentally different to ours and Zhang et al. (2023) approaches. We compare these 3 BMA approaches in Section 6.3.

The primary motivation for this work is to mitigate the risk of potentially counterintuitive recommendations and estimations that a dose-finding model might imply. Clinicians’ trust is an essential aspect of running real-world adaptive clinical trials with more advanced statistical modelling (Mozgunov et al. 2022). Even if a clinical trial design has good statistical properties, as confirmed via a simulation study, the design may not be implemented in practice if its recommendations are not aligned with clinical reasoning or the totality of the trial data (Yap et al. 2017). A lack of trust in the design can lead to the recommendations given by the model being overruled or disregarded, even in the presence of evidence that supports the model’s decision.

Escalation coherency (Cheung 2005; Tighiouart and Rogatko 2010; Wheeler 2018) has been proposed as a formal means of evaluating whether the escalation decisions made by an adaptive model are in line with observed outcomes. In particular, this type of coherency implies that designs that recommend dose escalation following a DLT or de-escalation after observing no DLT are not desirable. Beyond escalation decisions, adaptive models can be used to inform Dose Review Committee’s (also called Safety Review Committee’s) by providing an adequate summary of the dose toxicity risk across dose-combinations. In this case, the toxicity risk estimates themselves rather than the escalation recommendations are used to guide decisions. Hence, it is crucial that the estimates are reliable and intuitive. That is, that they are aligned with the clinical understanding of the increasing toxicity risk with the dose. This motivates the concept of *estimation coherency*, proposed in this article, which evaluates the consistency and interpretability of toxicity estimates in light of sequential trial observations and known dose relationships.

The objective of this article is 2-fold: (i) to formally define the POCRM based on model averaging and comprehensively evaluate its statistical properties in a simulation study; and (ii) to define the concept of estimation incoherence, emphasize its impact on the real-life implementation, and to demonstrate how BMA can tackle this problem for the POCRM. The general framework of the Bayesian CRM is outlined along with the POCRM and the novel method BMA-POCRM in Section 2. Section 3 provides clear motivation for the development of BMA-POCRM. Here, the novel concept of estimation coherency is introduced, which measures the consistency of dose toxicity estimate updates with respect to the given dose toxicity orderings. Furthermore, we provide a case study of the performance of BMA-POCRM and POCRM on real trial data in Section 4. In Section 5, theoretical results for estimation coherency under $ 2\times 2 $ grid sizes are given for the POCRM and BMA-POCRM. Together, the evidence from the case study, theory, and further simulation results in Section 6 show that BMA-POCRM improves the accuracy, safety and operating characteristics with more intuitive escalation and de-escalation decisions. Finally, a discussion and analysis of the results are presented in Section 7.

## 2. METHODS

### 2.1. General framework

Consider a setting with a partial ordering corresponding to $ M $ simple orderings and $ K $ dose levels, $ \{d_{1},\ldots, d_{K}\} $. Following a framework similar to that set by the original CRM (O’Quigley et al. 1990), let $ X_{j} $ be the dose level assigned to the $ j $-th patient where $ x_{j}\in\{d_{1},\ldots, d_{K}\} $ and let $ Y_{j} $ be a binary random variable for whether patient $ j $ experiences a DLT.

For a particular ordering $ m\in\{1 , \ldots, M\} $, the risk of DLT at $ d_{k}\in\{d_{1},\ldots, d_{K}\} $ is modelled as,


\begin{align*}\hat{R}(d_{k})=\Pr[Y_{j}=1|X_{j}=d_{k}]=\psi_{m}(d_{k},a_{m}),\end{align*}

where $ \hat{R}(d_{k}) $ is the estimated risk of DLT at $ d_{k} $, $ \psi_{m} $ is the working model under ordering $ m $ (Wages et al. 2011), with ordering-specific parameter $ a_{m} $. A wide range of specifications can be selected for the working model, each with their own associated assumptions regarding the dose-toxicity relationship and parameter estimation approaches (Cheung and Chappell 2002). A necessary assumption of the working model is that the relationship between dose level and dose toxicity is monotonic under a given simple ordering. This allows for multiple models to be specified based on the defined set of simple orderings, with each model corresponding to a specific ordering of doses.

The dose level for the next cohort of patients is allocated by minimizing the difference between estimated risk of DLT and the TTR, which can be expressed as the following criterion,


(1) \begin{align*} x_{j+1}=\mathop{\rm arg\, min}_{d_{k}}|\hat{R}(d_{k})-\theta|,\end{align*}

where $ \theta $ is the TTR. By repeating this estimation-minimization process until the stopping conditions are satisfied, an estimate for the true MTD is obtained, which is the dose recommended by the model following the final cohort of patients.

### 2.2. Continual reassessment method for partial ordering

Under the Bayesian framework of the POCRM, a potentially ordering-specific prior distribution for the model parameters, $ f_{m}(a_{m}) $, and a prior probability for each ordering $ p(m) $ where $ \sum_{m\,=\,1}^{M}p(m)=1 $ and $ p(m)\geq 0\;\forall m $ is required.

Since $ Y $ is binary, the likelihood takes the form of a Bernoulli random variable, where each patient in a cohort either experiences or does not experience a DLT. The observed data up to patient $ j $ is defined as $ \Omega_{j}=\{x_{1},y_{1},\ldots, x_{j},y_{j}\} $. This gives the following likelihood under ordering $ m $ after the inclusion of $ j $ patients in the trial,


(2) \begin{align*} L_{m}(a_{m}|\Omega_{j})=\prod_{l=1}^{j}\{\psi_{m}(x_{l},a_{m})\}^{y_{l}}\{1-\psi_{m}(x_{l},a_{m})\}^{1-y_{l}},\end{align*}

where $ x_{l} $ is the dose allocated to patient $ l $, and $ y_{l} $ is the binary variable denoting whether patient $ l $ experiences a DLT and $ \Omega_{j} $ contains the paired patient data $ (x_{l},y_{l}) $. Given the likelihood, the posterior density for the parameter $ a_{m} $ under the $ m $-th model is given by


(3) \begin{align*} f_{m}(a_{m}|\Omega_{j})=\frac{L_{m}(a_{m}|\Omega_{j})f_{m}(a_{m})}{\int_{\mathcal{A}}L_{m}(a_{m}|\Omega_{j})f(a_{m})da_{m}}.\end{align*}

That is, each model will have a unique posterior distribution for $ a_{m} $, as each model implies a unique ordering of doses. The posterior probabilities for each ordering are also obtained via Bayes’ rule as follows,


(4) \begin{align*} p(m|\Omega_{j})=\frac{p(m)\int_{\mathcal{A}}L_{m}(a_{m}|\Omega_{j})f_{m}(a_{m})da_{m}}{\sum_{m^{\prime}=1}^{M}p(m^{\prime})\int_{\mathcal{A}}L_{m^{\prime}}(a_{m}|\Omega_{j})f(a_{m})da_{m}}.\end{align*}

The model used for dose allocation is selected by maximizing the posterior model probabilities,


(5) \begin{align*} m^{*}=\mathop{\rm arg\, max}_{m}p(m|\Omega_{j}),\end{align*}

which results in a single partial ordering being selected for downstream estimation. The posterior density corresponding to this model is then used to estimate a posterior mean for parameter $ a_{m} $,


(6) \begin{align*}\hat{a}_{m^{*}}=\int_{\mathcal{A}}a_{m}f_{m^{*}}(a_{m}|\Omega_{j})da_{m},\end{align*}

which can be plugged directly into the working model to obtain an estimate for the risk of DLT for the $ k $-th dose,


(7) \begin{align*}\hat{R}(d_{k})=\psi_{b}(d_{k},\hat{a}_{m^{*}}),\end{align*}

from which the next dose is allocated using the criterion expressed in Equation (1).

### 2.3. Bayesian model averaging POCRM (BMA-POCRM)

Suppose that rather than selecting a single model or ordering for each dose allocation in the trial, all orderings are taken into account before making the next decision on dose allocation. BMA (Raftery et al. 1997) would allow for estimates for probability of toxicity under multiple orderings to be combined for a single probability across several models. This is done by combining the available posterior information on the set of parameters $ a_{m} $ and the $ M $ model probabilities. BMA can be applied such that it accounts for the full posterior distribution of the set of parameters $ a_{m} $. This allows for incorporating uncertainty in $ a_{m} $ and partial orderings before obtaining a point estimate of the risk of DLT of each dose level.

By applying a change of variables to $ f_{m}(a_{m}|\Omega_{j}) $, the working model for $ R(d_{k}) $ is expressed as a probability distribution. Although $ R(d_{k}) $ is a useful expression for interpretation, $ R(d_{k}) $ for each $ k $ is estimated independently of the others, hence, $ R(d_{k}) $ is not a function of $ d_{k} $ but a function of $ a_{m} $ since $ R(d_{k})=\psi_{m}(d_{k},a_{m}) $. The change in variable is $ R(d_{k})=\psi_{m}(d_{k},a_{m}) $ such that from the combined posterior distribution the following probability distribution function for $ R(d_{k}) $ can be obtained. We compute the density for $ R(d_{k}) $ directly from the ordering-specific densities as follows:


(8) \begin{align*} f_{m}(R(d_{k})|\Omega_{j})=\left|\frac{d\psi^{-1}_{m}(d_{k},R(d_{k}))}{dR(d_{k})}\right|f_{m}(a_{m}|\Omega_{j}).\end{align*}

Applying BMA, we obtain the combined posterior distribution for the risk of toxicity,


(9) \begin{align*} g(R(d_{k})|\Omega_{j})=\sum_{m=1}^{M}p(m|\Omega_{j})f_{m}(R(d_{k})|\Omega_{j}),\end{align*}

which is independent of ordering. The expectation for the risk of toxicity under dose $ d_{k} $ is then,


(10) \begin{align*}\mathbb{E}[R(d_{k})]=\int_{0}^{1}R\left(d_{k})\; g(R(d_{k})|\Omega_{j}\right)\; d\{R(d_{k})\}.\end{align*}

Finally, dose allocation is carried out under this framework by setting $ \hat{R}(d_{k})=\mathbb{E}[R(d_{k})] $ and applying the criterion described in Section 2.1.

## 3. COHERENCY IN THE PRESENCE OF PARTIAL ORDERING

### 3.1. Defining estimation coherency

Coherence in Phase I clinical trials is a useful concept for assessing the theoretical qualities of trial methodology. Throughout this article, coherence, which is defined based on escalation and de-escalation behaviour of a dose-finding model, is referred to as *escalation coherence*. In practice, an escalation coherent design will benefit patient safety as it reduces the likelihood of assigning an overly toxic dose to a patient or cohort but will also ensure that the maximal dose level considered safe is administered. Expanding to the drug combination setting, Park and Liu (2020) introduce definitions of strong and weak coherency, both of which rely on evaluating the characteristics of escalation and de-escalation to define coherency. A definition of escalation coherency which is equally applicable to both single-agent and dual-agent combination trials is also presented.

There are 2 sets of doses that are relevant to escalation coherency. Let $ \mathcal{E}_{n} $ and $ \mathcal{D}_{n} $ contain the candidate dose levels for escalation and de-escalation, respectively, for dose allocation $ X_{n} $.

Definition 1 (Escalation Coherency). A design is coherent in dose escalation if $ \Pr[X_{n\,+\,1}\in\mathcal{E}_{n}|Y_{n}=1]=0 $ for $ n\,=\,1 , \ldots, d-1 $ and is coherent in dose de-escalation if $ \Pr[X_{n\,+\,1}\in\mathcal{D}_{n}|Y_{n}=0]=0 $ for $ n\,=\,1 , \ldots, d-1 $. A design is escalation coherent if dose escalation and de-escalation are coherent.

This definition emphasizes the sole concept of dose selection. Whereas, during the administration of a real-world trial, there is frequent interface between domain expert and dose-escalation model. Domain experts use both the given toxicity estimates and the recommended next dose to make a final escalation decision. This is exceedingly relevant to the combination setting where there could be several combinations to choose from at any given point in the trial. To address the need for both toxicity estimates and dose escalation recommendations to be coherent, the following specification of *estimation coherency* is introduced.

Definition 2 (*Two-sided Estimation Coherency*). *Suppose we are given a partial ordering over a set of doses, from which $ M $ total orderings are derived. Let $ I_{m}(d_{i}) $ denote the index of dose $ d_{i} $ in the $ m $-th ordering. If dose $ d_{i} $ is more toxic than dose $ d_{j} $ in ordering $ m $, then $ I_{m}(d_{j}) < I_{m}(d_{i}) $, and vice versa.*

*For each dose $ d_{i} $, define:*

• *The set of universally less toxic doses:*
 
  \begin{align*}\nu_{i}=\left\{d_{j}:I_{m}(d_{j}) < I_{m}(d_{i})\;\;\forall m\right\},\end{align*}
• *The set of universally more toxic doses:*
 
  \begin{align*}\xi_{i}=\left\{d_{j}:I_{m}(d_{j}) > I_{m}(d_{i})\;\;\forall m\right\}.\end{align*}

*A design is said to be* ***2-sided estimation coherent*** *if it satisfies:*

(i) *Following no DLT at dose* $ d_{i} $*, the estimated toxicity risk* $ \hat{R}(d_{j}) $  *decreases for all* $ d_{j}\in\nu_{i}\cup\xi_{i} $.
(ii) *Following a DLT at dose* $ d_{i} $*, the estimated toxicity risk* $ \hat{R}(d_{j}) $  *increases for all* $ d_{j}\in\nu_{i}\cup\xi_{i} $.

Throughout this work, we refer to 2-sided estimation coherency simply as *estimation coherency*. We also define a less strict subclass of estimation coherency called *one-sided estimation coherency* as follows:

Definition 3 (*One-sided Estimation Coherency*). *Following the setup and notation from Definition 2, a design is said to be* ***1-sided estimation coherent*** *if it satisfies:*
 

(i) *Following no DLT at dose* $ d_{i} $*, the estimated toxicity risk* $ \hat{R}(d_{j}) $  *decreases for all* $ d_{j}\in\nu_{i} $,
(ii) *Following a DLT at dose* $ d_{i} $*, the estimated toxicity risk* $ \hat{R}(d_{j}) $  *increases for all* $ d_{j}\in\xi_{i} $.

BMA mitigates estimation incoherence by softening transitions between competing orderings of toxicity. Instead of committing to a single ordering at each step, BMA performs a weighted averaging of the toxicity estimates across all plausible orderings according to their posterior probabilities. This smoothing effect dampens abrupt shifts in toxicity estimates that can occur when switching between models, thereby reducing the likelihood of incoherent updates following new observations. It will be shown in the theoretical results (Section 5) and simulation study (Section 6) below that all estimation incoherencies of the POCRM occur when there is a change in the selected ordering.

Both 2-sided and 1-sided estimation coherencies act as an essential check for dose-finding models. By ensuring a model coincides with both the prior knowledge implied by the partial ordering and most recently gained information, it is able to detect changes which are potentially misaligned with the assumption of increasing toxicity within a dose and may endanger the trustworthiness of the model.

Another quantity associated with estimation coherency is the magnitude of changes in toxicity estimates. Large changes in toxicity estimates indicate large changes in model belief, and whilst these changes do not harm patients directly, they can be undesirable to clinicians overseeing a real-world trial. We comment on these types of changes where relevant.

### 3.2. Illustrative example

The POCRM is particularly vulnerable to estimation incoherencies. Here, a specific example of estimation incoherency under the POCRM is explored.

Consider the $ 3\times 2 $ drug combination design shown in Table 1. The model skeleton for both the POCRM and BMA-POCRM is generated using the getprior function (Cheung 2019) with $ \delta{\mathrm{(halfwidth)}}=0.02 $ and $ \nu{\text{(prior MTD)}}=2 $. These parameters were selected based on a hyperparameter tuning process as described in Section S.2. There are several candidates for a working model $ \psi_{m}(d_{k},a_{m}) $. Throughout this work, we use the parametrization of the power model given in Wages et al. (2011) with the following form,


\begin{align*}\psi_{m}(d_{k},a_{m})=\alpha_{mk}^{a},\;\;\;\; k=1 , \ldots, K\end{align*}

where $ a_{m}\in[0 , \infty) $ and $ 0 \lt\alpha_{m1} \lt\ldots, \lt\alpha_{mK} $ is the probability skeleton, which represents the prior estimates of dose toxicity at each dose level under ordering $ m $. The prior distribution of $ a_{m} $ is Normal with mean 0 and variance 1.34 as suggested by Wages et al. (2011). Furthermore, we also explored different variance parameters for the working model, however, in Section S.3, we show that the selected variance of the normal prior has no impact on model performance. We use a cohort size of 1 with a TTR of 0.4. Applying the partial ordering specification recommended by Wages and Conaway (2013), the following 6 simple orderings are used for both POCRM and BMA-POCRM, of which 5 are unique,


\begin{align*} m=1 : \; d_{1}\rightarrow d_{2}\rightarrow d_{3}\rightarrow d_{4}\rightarrow d_{5}\rightarrow d_{6},\\m=2 : \; d_{1}\rightarrow d_{3}\rightarrow d_{5}\rightarrow d_{2}\rightarrow d_{4}\rightarrow d_{6},\\m=3 : \; d_{1}\rightarrow d_{3}\rightarrow d_{2}\rightarrow d_{5}\rightarrow d_{4}\rightarrow d_{6},\\m=4 : \; d_{1}\rightarrow d_{2}\rightarrow d_{3}\rightarrow d_{4}\rightarrow d_{5}\rightarrow d_{6},\\m=5 : \; d_{1}\rightarrow d_{2}\rightarrow d_{3}\rightarrow d_{5}\rightarrow d_{4}\rightarrow d_{6},\\m=6 : \; d_{1}\rightarrow d_{3}\rightarrow d_{2}\rightarrow d_{4}\rightarrow d_{5}\rightarrow d_{6}.\end{align*}

This set of orderings is the complete set of possible orderings, assuming that dose-toxicities increase monotonically only where the dose level increases in only one drug of the combination. At the start of the trial, the a priori probability for each ordering is equal.

From these simple orderings one can obtain the sets of interest $ \nu_{i} $ and $ \xi_{i} $ for each dose as shown for each dose level in Table 2. The sets used to check for estimation incoherencies are composed as follows. For $ d_{2} $, $ \nu_{2}=\{d_{1}\} $ and $ \xi_{2}=\{d_{4},d_{6}\} $. This is obtained by considering that $ d_{1} $ is less toxic than $ d_{2} $ under every simple ordering, and both $ d_{4} $ and $ d_{6} $ are more toxic than $ d_{2} $ under every simple ordering. These sets exist for each dose level and can be used following each Bayesian probability update to detect any estimation incoherencies.

**Table 2.** Sets for detecting estimation incoherencies, where $ \nu_{i} $ is the set of doses always less toxic than dose $ i $ and $ \xi_{i} $ is the set of doses always more toxic than dose $ i $.

Dose level, $ i $	$ \nu_{i} $	$ \xi_{i} $
1	$ \emptyset $	$ \{d_{2},d_{3},d_{4},d_{5},d_{6}\} $
2	$ \{d_{1}\} $	$ \{d_{4},d_{6}\} $
3	$ \{d_{1}\} $	$ \{d_{4},d_{5},d_{6}\} $
4	$ \{d_{1},d_{2},d_{3}\} $	$ \{d_{6}\} $
5	$ \{d_{1},d_{3}\} $	$ \{d_{6}\} $
6	$ \{d_{1},d_{2},d_{3},d_{4},d_{5}\} $	$ \emptyset $

### 3.3. Applying POCRM with model selection

1,000 simulations were initiated with the first estimation incoherency identified in the second simulated trial at the induction of cohort 12. At this point in the trial, the posterior ordering probabilities are shown in Table 3. The dose allocations and DLTs observed up to and including cohort 11 are as follows,


\begin{align*}\mathbf{n}^{[11]}&=(1,0,1,6,2,1),\\\mathbf{y}^{[11]}&=(0,0,0,3,1,1),\end{align*}

where the $ i $-th entry in $ \mathbf{n}\in\mathbb{Z}^{6} $ is the number of patients assigned to the $ i $-th dose level, $ d_{i} $, and $ \mathbf{y}\in\mathbb{Z}^{6} $ is the number of patients that experienced a DLT after being assigned dose level $ d_{i} $. As seen in Table 3, following the induction of cohort 11, $ d_{2} $ is recommended as the next dose by POCRM. Hence, cohort 12 is inducted and administered $ d_{2} $, which yields the following allocation vectors,


\begin{align*}\mathbf{n}^{[12]}&=(1,1,1,6,2,1),\\\mathbf{y}^{[12]}&=(0,0,0,3,1,1),\end{align*}

where no new DLT is observed for $ d_{2} $.

**Table 3.** Model probabilities and dose-toxicity estimates for POCRM and BMA-POCRM dose-escalation frameworks in a setting with a TTR of 0.4.

Method	Cohort	$ p(\cdot|x): $ (posterior model probability)					
		$ m=1 $	$ m=2 $	$ m=3 $	$ m=4 $	$ m=5 $	$ m=6 $
POCRM	11	0.1568	0.1497	0.1878	0.1568	0.1582	0.1906
	12	0.1743	0.1091	0.1840	0.1743	0.1840	0.1743
BMA-POCRM	11	0.1568	0.1497	0.1878	0.1568	0.1582	0.1906
	12	0.1743	0.1091	0.1840	0.1743	0.1840	0.1743

Method	Cohort	$ R(\cdot): $ (estimated probability of toxicity)					
		$ d_{1} $	$ d_{2} $	$ d_{3} $	$ d_{4} $	$ d_{5} $	$ d_{6} $
POCRM	11	0.0672	0.3261	0.1756	**0.4859**	0.6282	0.7412
	12	0.0331	0.1114	0.2432	**0.5562**	0.4023	0.6853
BMA-POCRM	11	0.0802	0.2671	0.2371	0.5247	0.5019	0.7111
	12	0.0654	0.2281	0.2210	0.4975	0.4860	0.6933

The recommended dose levels and selected orderings following each cohort are shown with an underline. Incoherencies are shown in bold. *Note*: Despite, the recommended dose-level given by BMA-POCRM being $ d_{5} $ following cohort 11, $ d_{2} $ is treated as the recommended dose to maintain consistency for comparison of POCRM and BMA-POCRM.

Recalling the set of doses with known toxicity relative to $ d_{2} $ in Section 3.2, since no DLT is observed at $ d_{2} $ the dose-toxicity estimates for all dose levels in $ \nu_{2}\cup\xi_{2}=\{d_{1},d_{4},d_{6}\} $ are expected to decrease. However, in Table 3, an increase in the toxicity estimate for $ d_{4} $ from 0.49 to 0.56 is observed. This is a change of +0.07 despite there being no information gained from the previous cohort that indicates a greater toxicity of $ d_{4} $. Throughout the coming analyses, the occurrence of estimation incoherencies is considered as a key operating characteristic of the methods being studied.

This example illustrates the often illogical changes in toxicity estimates observed under the POCRM. Since toxicity estimates guide clinicians and affect the next allocated dose, it is crucial that changes in these toxicity estimates are robust to scrutiny.

### 3.4. Applying BMA-POCRM

Again, under this setting, the dose-toxicity estimates for all dose levels in $ \nu_{2}\cup\xi_{2}=\{d_{1},d_{4},d_{6}\} $ are expected to decrease or remain the same as there was no DLT observed for $ d_{2} $. Returning to the results presented in Table 3 for BMA-POCRM, for all doses in $ \nu_{2}\cup\xi_{2} $ the toxicity estimate decreases. Conversely to POCRM, BMA-POCRM remains coherent with respect to estimation coherency in this case. Furthermore, POCRM exhibits several large changes in toxicity estimates here. The estimates corresponding to doses $ d_{2} $ and $ d_{5} $ change by $ -0.2147 $ and $ -0.2259 $, respectively. For BMA-POCRM, these doses change by $ -0.039 $ and $ -0.0159 $, respectively, where they also correspond to the greatest change in toxicity estimates among the candidates. In Section S.8, we demonstrate empirically that the POCRM is estimation incoherent due to changes in the selected dose ordering. Specifically, it is demonstrated that this is mitigated by the BMA-POCRM since it does not select a single ordering model.

## 4. CASE STUDY

To consider the performance of BMA-POCRM in comparison to POCRM in a real-world setting, we apply these methods to a Phase I study dosing patients combinations of neratinib and temsirolimus (Gandhi et al. 2014). This trial involved 52 patients treated on 12 doses in a 4-by-4 grid of possible neratinib-temsirolimus combinations with a TTR of $ \theta\,=\,1/3 $. The remaining 4 doses in the grid were never assigned to patients in the original trial. A DLT is defined as an inability to maintain the prescribed dose for the first 28 days of treatment due to treatment-related toxicity. The 2 initial cohorts consisted of 2 patients each were enrolled simultaneously with: (i) 160 mg of neratinib/15 mg of temsirolimus; and (ii) 120 mg of neratinib/25 mg of temsirolimus.

We apply BMA-POCRM using the same parameters and orderings as specified in Section 3.2. The full trial data and results used for these simulations can be found in Section S.5.

The aim here is to identify incoherencies and large changes in toxicity estimates. By showing that these occur in real-world trials we aim to further motivate the use of BMA-POCRM in practice.

### 4.1. Data generation

To allow for a fair evaluation of model-guided dose escalation, we use the original trial data to conduct a case study according to the scheme outlined by Barnett et al. (2024). We define a fixed set of 52 patient dose responses for each dose. Here, we denote the number of patients assigned to dose $ j $ by $ n_{j} $ and the number of observed DLTs under dose $ j $ by $ y_{j} $.

To define a fixed set of 52 patient dose responses for each dose we take the first $ n_{j} $ responses to be a random permutation of responses from the original study. The remaining $ 52-n_{j} $ responses are generated from a Beta$ (1+y_{j},1+n_{j}-y_{j}) $ distribution by first sampling a probability of DLT and sampling a binary response from a Bernoulli distribution with the given probability of DLT. Where no patients are assigned to a dose combination in the real study, probabilities are generated from a Beta$ (3,3) $ distribution instead. As each method allocates a dose to each cohort, the ordered set of responses corresponding to each dose is used to determine treatment response sequentially. This ensures that the $ n $th patient allocated to each dose, regardless of method of dose escalation used, will have the same response to treatment.

### 4.2. Results

**Fig. 1.**
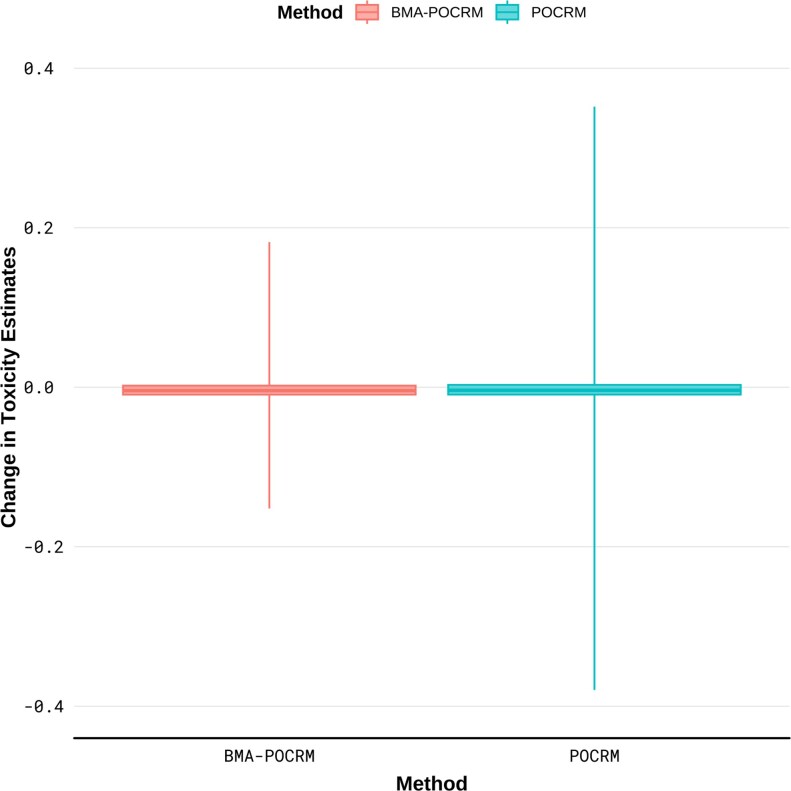
Distribution of the magnitude of changes in toxicity estimates in the motivating trial simulation. Whiskers indicate range of maximum and minimum values observed.

Figure 1 shows that the range of magnitude of changes in toxicity estimate for POCRM is larger than for BMA-POCRM. The range is shown to be [--0.17, 0.18] and [--0.37, 0.35] for BMA-POCRM and POCRM, respectively.

**Fig. 2.**
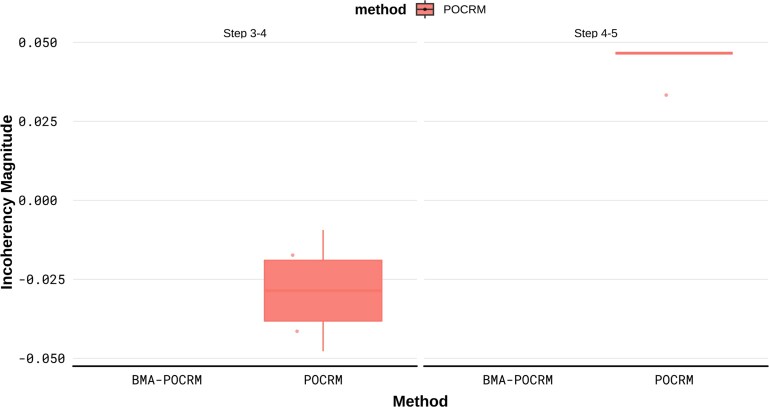
Incoherencies that occurred in motivating trial.

Table 4 shows an example of a cohort where the large change in toxicity estimate is particularly great. After assigning $ d_{8} $ to cohort 3, we observe a DLT, which leads to several incoherencies and large changes in toxicity estimates for the estimates that are to be used to assign doses to cohort 4. For POCRM, we observe 2 changes that are $ > 25 $, for $ d_{4} $ and the administered dose $ d_{8} $. We also observe 2 estimate incoherencies for doses $ d_{5} $ and $ d_{6} $, which we know are less toxic than $ d_{8} $ according to the partial orderings corresponding to this trial setting. For this cohort, the toxicity estimates given by BMA-POCRM do not yield any incoherencies or large changes as is true for the remainder of the motivating trial, as shown in Fig. 2.

**Table 4.** Example of a sudden change in toxicity estimates under POCRM in the motivating trial.

		$ R(\cdot): $ (estimated probability of toxicity)													
Method	Cohort	$ d_{1} $	$ d_{2} $	$ d_{3} $	$ d_{4} $	$ d_{5} $	$ d_{6} $	$ d_{7} $	$ d_{8} $	$ d_{9} $	$ d_{10} $	$ d_{11} $	$ d_{12} $	$ d_{\rm sel} $	$ y_{\rm sel} $
	3	0.00	0.00	0.01	**0.04**	**0.08**	**0.15**	0.23	**0.33**	0.43	0.53	0.61	0.69	$ d_{8} $	1
POCRM	4	0.00	0.01	0.07	**0.32**	**0.03**	**0.14**	0.42	**0.68**	0.22	0.51	0.75	0.60	–	–
	3	0.01	0.04	0.13	0.25	0.06	0.13	0.29	0.48	0.18	0.36	0.52	0.41	$ d_{10} $	0
BMA-POCRM	4	0.01	0.02	0.09	0.20	0.03	0.08	0.22	0.41	0.11	0.27	0.44	0.32	–	–

Any sudden changes (ie those greater than 0.25) and any incoherencies are shown in bold.

Figure 2 shows that incoherencies only occurred for POCRM in the motivating trial, whilst BMA-POCRM solved this problem completely. The incoherencies shown for steps 3–4 correspond to the results in Table 4. The full dose allocations can be found in Section S.5.

## 5. CONDITIONS FOR ESTIMATION COHERENCY

This section provides sufficient conditions for the estimation coherency of the POCRM with model selection (POCRM) and the BMA-POCRM in the setting of $ 2\times 2 $ combinations, shown in Table 5. Throughout this section, coherence of the CRM defined in Cheung (2005) is assumed. Suppose the 2 orderings


\begin{align*} 1 : \quad d_{1}\to d_{2}\to d_{3}\to d_{4}, \ 2 : \quad d_{1}\to d_{3}\to d_{2}\to d_{4},\end{align*}

are both included with equal prior ordering probabilities $ p(1)=p(2)=1/2 $. Consider the one-parameter power model $ \psi_{m}(d_{k},a_{m})=\alpha_{m, k}^{a} $, where $ (\alpha_{m, k})_{k\,=\,1}^{K} $ is a permutation of the monotonically increasing toxicity skeletons $ \boldsymbol{\alpha}=(\alpha_{1},\ldots , \alpha_{k}) $ under ordering $ m $. Suppose patients are enrolled one at a time. Let $ \hat{a}_{m}^{(j)} $, $ m\,=\,1,2 $, be the posterior estimate of the model parameter $ a_{m} $ after the first $ j $ patients.

**Table 5.** $ 2\times 2 $ setting resulting in 4 dose levels.

		Drug B	
		1	2
Drug A	2	$ \boldsymbol{d}_{\bf 3} $	$ \boldsymbol{d}_{\bf 4} $
	1	$ \boldsymbol{d}_{\bf 1} $	$ \boldsymbol{d}_{\bf 2} $

By the estimation coherency of the CRM (Cheung 2005), the POCRM is estimation-coherent from the $ j $th to the $ (j\,+\,1) $th patient if the selected ordering is the same. On the other hand, if, without loss of generality, the selected ordering changed from ordering 1 to ordering 2, then, after observing a non-DLT at $ d_{1} $, there is no guarantee that the estimated DLT probability at $ d_{2}\in\nu_{1} $ will decrease. This is formalized in Lemma 1. A sufficient condition for the coherence of the POCRM is summarized in Theorem 2 below.

The proofs of all theoretical results are provided in Section S.6 of the Supplementary Materials.

Lemma 1. *Given the regularity conditions required for the coherency of the CRM, as specified in*  Cheung (2005), *estimation-incoherence of the POCRM happens only after a change of the selected ordering.*

Theorem 2 (POCRM coherence). *For $ 2\times 2 $ combinations with both orderings included with equal prior probabilities. The POCRM is estimation coherent given the sufficient condition*
 

(11) \begin{align*}\alpha_{3}^{\hat{a}_{2}^{\min}}\leq\alpha_{2}^{\hat{a}^{\max}_{1}},\qquad\alpha_{3}^{\hat{a}_{1}^{\min}}\leq\alpha_{2}^{\hat{a}^{\max}_{2}},\end{align*}

*where $ \hat{a}_{1}^{\min}=\min\mathcal{A}_{1} $, $ \hat{a}_{1}^{\max}=\max\mathcal{A}_{1} $, $ \hat{a}_{2}^{\min}=\min\mathcal{A}_{2} $, $ \hat{a}_{2}^{\max}=\max\mathcal{A}_{2} $, and $ \mathcal{A}_{1}=\{\log R_{1}/\log\alpha_{1} $, $ \log R_{2}/\log\alpha_{2} $, $ \log R_{3}/\log\alpha_{3} $, $ \log R_{4}/\log\alpha_{4}\} $, $ \mathcal{A}_{2}=\{\log R_{1}/\log\alpha_{1} $, $ \log R_{2}/\log\alpha_{3} $, $ \log R_{3}/\log\alpha_{2} $, $ \log R_{4}/\log\alpha_{4}\} $, and $ R_{k} $ is the true toxicity probability at $ {d}_{k} $.*

Similarly, a set of sufficient condition for the BMA-POCRM to be estimation coherent is summarized in Theorem 3 below.

Theorem 3 (BMA-POCRM estimation-coherence). *For $ 2\times 2 $ combinations, when both orderings are included with equal prior ordering probabilities. A sufficient condition for the estimation-coherence is*
 

(12) \begin{align*}\begin{split}\frac{1}{1-\log\alpha_{1}}\leq&\min\left\{\frac{\alpha_{3}^{\hat{a}_{2}^{\min}}\left(\alpha_{3}^{U_{1}}-1\right)} {1-\alpha_{2}^{\hat{a}_{1}^{\min}}/\alpha_{3}^{\hat{a}_{2}^{\max}}},\frac{\alpha_{3}^{\hat{a}_{1}^{\min}}\left(\alpha_{3}^{U_{2}}-1\right)}{1-\alpha_{2} ^{\hat{a}_{2}^{\min}}/\alpha_{3}^{\hat{a}_{1}^{\max}}},\right\},\nonumber\\\frac{1}{1-\log\alpha_{4}}\leq&\min\left\{\frac{\alpha_{3}^{\hat{a}_{2}^{\min}}\left(\alpha_{3}^{U_{3}}-1\right)}{1-\alpha_{2} ^{\hat{a}_{1}^{\min}}/\alpha_{3}^{\hat{a}_{2}^{\max}}},\frac{\alpha_{3}^{\hat{a}_{1}^{\min}}\left(\alpha_{3}^{U_{4}}-1\right)}{1-\alpha_{2}^{\hat{a}_{2}^{\min}}/\alpha_{3}^{\hat{a}_{1}^{\max}}},\right\},\end{split}\end{align*}

*where*
 

\begin{align*} U_{1}=\frac{1-2\log\alpha_{1}}{1-\log\alpha_{1}}\hat{a}^{\max}_{2}-\frac{1}{(1-\log\alpha_{1})^{2}},& U_{2}=\frac{1-2\log\alpha_{1}}{1-\log\alpha_{1}}\hat{a}^{\max}_{1}-\frac{1}{(1-\log\alpha_{1})^{2}},\\U_{3}=\frac{1-2\log\alpha_{4}}{1-\log\alpha_{4}}\hat{a}^{\max}_{2}-\frac{1}{(1-\log\alpha_{4})^{2}},& U_{4}=\frac{1-2\log\alpha_{4}}{1-\log\alpha_{4}}\hat{a}^{\max}_{4}-\frac{1}{(1-\log\alpha_{4})^{2}},\end{align*}

$ \hat{a}_{1}^{\min}=\min\mathcal{A}_{1} $*, $ \hat{a}_{1}^{\max}=\max\mathcal{A}_{1} $, $ \hat{a}_{2}^{\min}=\min\mathcal{A}_{2} $, $ \hat{a}_{2}^{\max}=\max\mathcal{A}_{2} $, and $ \mathcal{A}_{1}=\{\log R_{1}/\log\alpha_{1} $, $ \log R_{2}/\log\alpha_{2} $, $ \log R_{3}/\log\alpha_{3} $, $ \log R_{4}/\log\alpha_{4}\} $, $ \mathcal{A}_{2}=\{\log R_{1}/\log\alpha_{1} $, $ \log R_{2}/\log\alpha_{3} $, $ \log R_{3}/\log\alpha_{2} $, $ \log R_{4}/\log\alpha_{4}\} $, and $ R_{k} $ is the true toxicity probability at $ {d}_{k} $.*

When there is a change of the selected ordering, the estimation coherence of the POCRM guarantees the estimation coherence of the BMA-POCRM, which is stated in Theorem 4. However, when the selected ordering is not changed, the POCRM is guaranteed to be coherent, whereas, it is still possible for the BMA-POCRM to be incoherent. An example of the BMA-POCRM being incoherent without a change of the selected ordering is given in Section S.8.

Theorem 4 (coherence of POCRM $ \Rightarrow $ coherence of BMA-POCRM). *When there is a change of the selected ordering from patient $ j $ to patient $ (j\,+\,1) $, $ j\,=\,1,2 , \ldots, n $, if the POCRM is estimation coherent, the BMA-POCRM is also estimation coherent.*

Finally, when the BMA-POCRM is estimation incoherent, the magnitude of the incoherence is bounded by the magnitude under the POCRM. This is stated in Theorem 5 below.

Theorem 5 (The magnitude of incoherence). *When neither the POCRM nor BMA-POCRM is coherent w.r.t. estimation, let $ M_{S} $ and $ M_{A} $ be the magnitude of incoherence under the POCRM and BMA-POCRM, respectively. Then*,

(13) \begin{align*} M_{A}\leq\frac{1}{(1-\log\alpha_{4})}M_{S}.\end{align*}

The above results imply that, for a $ 2\times 2 $ grid, the BMA-POCRM is less often incoherent than the POCRM, a result that is confirmed by simulations for other grid sizes in Section 6.

## 6. SIMULATION STUDY

### 6.1. Specification

A simulation study is necessary to evaluate and compare the operating characteristics for POCRM and BMA-POCRM in a large-scale setting. For this study, a 4-by-4 structure is selected in a 2 drug combination study. For each scenario, $ 10,000 $ trial simulations are run with a TTR of $ \theta\,=\,0.3 $ and a cohort size of 1 for 60 cohorts. As in Section 3, we use getprior to generate a the model skeletons with $ \delta\,=\,0.02 $ and $ \nu\,=\,2 $, as optimized in Section S.2 and the following orderings obtained as recommended by Wages and Conaway (2013),


\begin{align*} m=1 : \; d_{1}\rightarrow d_{2}\rightarrow d_{3}\rightarrow d_{4}\rightarrow d_{5}\rightarrow d_{6},\\m=2 : \; d_{1}\rightarrow d_{3}\rightarrow d_{5}\rightarrow d_{2}\rightarrow d_{4}\rightarrow d_{6},\\m=3 : \; d_{1}\rightarrow d_{3}\rightarrow d_{2}\rightarrow d_{5}\rightarrow d_{4}\rightarrow d_{6},\\m=4 : \; d_{1}\rightarrow d_{2}\rightarrow d_{3}\rightarrow d_{4}\rightarrow d_{5}\rightarrow d_{6},\\m=5 : \; d_{1}\rightarrow d_{2}\rightarrow d_{3}\rightarrow d_{5}\rightarrow d_{4}\rightarrow d_{6},\\m=6 : \; d_{1}\rightarrow d_{3}\rightarrow d_{2}\rightarrow d_{4}\rightarrow d_{5}\rightarrow d_{6}.\end{align*}

Furthermore, we examine the operating characteristics under various cohort sizes in Web Section S.4.

There are several candidates for a working model $ \psi_{m}(d_{k},a_{m}) $. As in the Illustrative Example in Section 3.2, we use the parametrization of the power model given in Wages et al. (2011) with a Normal prior on $ a_{m} $ with mean 0 and variance 1.34 as suggested by Wages et al. (2011). The selected variance of the normal prior has no impact on model performance as shown in Section S.3. This working model is applied for both POCRM and BMA-POCRM.

Five metrics are used to assess models in simulation trials according to recommendation accuracy, assignment accuracy, and patient safety. In this case, an overly toxic dose is chosen to be any dose that has a true probability of toxicity greater than 110% of the TTR.

(i) The proportion of correct selections (PCS) is the proportion of trials that recommended doses with a true probability of toxicity equal to the TTR.
(ii) The proportion of acceptable selections (PAS) is the proportion of trials that recommended a dose with a true probability of toxicity within $ [\theta-0.1 , \theta] $, where $ \theta $ is the TTR.
(iii) Proportion of trials that give overly toxic selections (POTS).
(iv) The number of patients treated at overly toxic doses (NPTOT).
(v) Finally, the estimation coherency of model estimates at the induction of each trial as defined in Definition 2.

The full specification for all 24 scenarios can be found in Section S.1. Scenarios with both symmetric and asymmetric true MTD locations are included. The number of accurate orderings present in the set of candidate orderings is also varied across scenarios. Those found in the left-most column of Table S1 have no correct orderings, implying that none of the orderings exactly match the specified probabilities of toxicity. Scenarios found in the middle column are satisfied by a single ordering in the list of candidate orderings. Finally, those found in the right-most column are equally satisfied by 2 candidate orderings.

### 6.2. Results

In this section, we account for potential numerical inaccuracies in computing the marginal integral in Equation (3) by restricting incoherency detection to those with absolute value greater than 0.001.

**Fig. 3.**
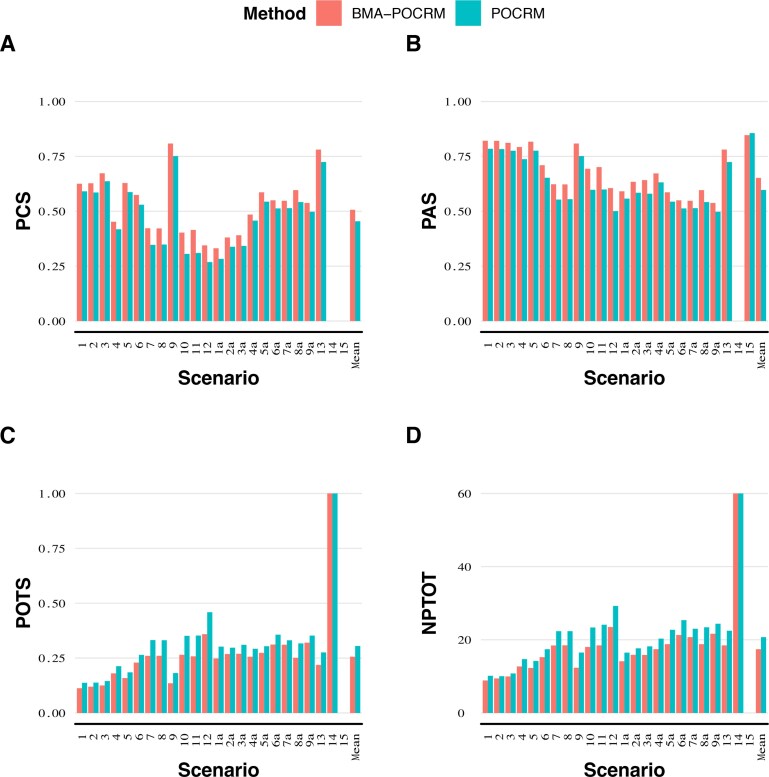
A) Arithmetic mean PCS across 10,000 repeated simulations with a cohort size 1 for each scenario. The mean performance across scenarios is also shown. B) Arithmetic mean PAS across 10,000 repeated simulations with a cohort size 1 for each scenario. The mean performance across scenarios is also shown. C) Arithmetic mean POTS across 10,000 repeated simulations with a cohort size 1 for each scenario. The mean performance across scenarios is also shown. D) Arithmetic mean PTOTD across 10,000 repeated simulations with a cohort size 1 for each scenario. The mean performance across scenarios is also shown.

Figure 3 illustrates model performance under all 24 scenarios, and provides an arithmetic mean performance across scenarios. BMA-POCRM exhibits better PCS and PAS across all scenarios but scenario 15, indicating that it more consistently selects desirable dose levels following the Phase I trial simulations.

BMA-POCRM leads to an average increase of 5.2% in PCS when compared to that of POCRM. Across all scenarios with non-zero PCS, there is an improvement in performance when applying BMA-POCRM. Considering scenarios 1-13, no scenario shows a lower difference than 2.77% in favour of BMA-POCRM. Scenario 12 shows the greatest discrepancy between the methods with BMA-POCRM exceeding POCRM by 10.7%. Likewise, investigating Fig. 3B, BMA-POCRM leads to an improvement by at least 3.34% and an average of 5.5%. For this measure, scenario 12 leads to the greatest difference in performance with 10.5%. Differences in the number of correct orderings between scenarios do not clearly favour either method and there is no consistent effect on performance associated with changes in the number of correct orderings.

The design matrices for scenarios 14 and 15 are such that there are only overly toxic doses and doses with a lower toxicity than the TTR, respectively. Under scenario 15, POCRM has a mean PAS of 85.61%, whilst BMA-POCRM has one of 84.67%. Nearly half of the doses in scenario 15 are considered acceptable by definition. Noting that these doses are in the lower triangular of the combination matrix, these are the most toxic doses under both drugs. This suggests that BMA-POCRM is the more conservative approach to dose escalation.

The safety of BMA-POCRM is further supported by Fig. 3C and D, where it recommends overly toxic doses in a smaller proportion of trials and allocates overly toxic doses to fewer patients, respectively. This trend is maintained throughout all scenarios and the mean POTS is 4.89% lower for BMA-POCRM than POCRM. Scenario 2 exhibits the smallest difference between methods in terms of POTS with a 1.84% decrease in the number of overly toxic selections with BMA-POCRM. In scenario 12, BMA-POCRM leads to a 10% reduction in the number of overly toxic doses. Figure 3D shows that on average, at least one cohort less will be assigned an overly toxic dose.

**Fig. 4.**
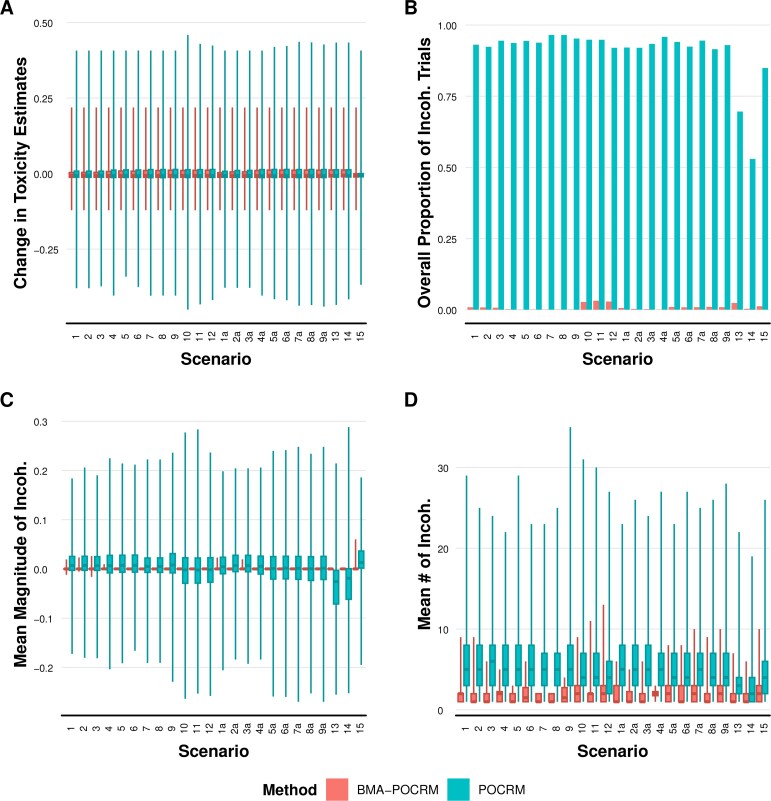
A) Distribution of changes in dose toxicity estimates following the induction of each cohort for simulated trials with cohort size 1. B) Proportion of trials where at least one incoherent estimate is observed. C) Magnitude of incoherent changes in toxicity estimates. D) Mean number of cohorts with at least one observed incoherency. *Note*: Only estimation incoherencies that exceed 0.001 are considered.

Figure 4A shows the magnitude and direction of changes in toxicity estimates across all trial steps in the simulated trials. From Fig. 4A, BMA-POCRM also has less dispersed estimates for each scenario.

The frequency of incoherencies within simulated trials is shown in Fig. 4B. The previous definition of estimation incoherency is here applied to evaluate the estimation operating characteristics of POCRM and BMA-POCRM. In all scenarios but 13, 14, and 15, POCRM leads to at least 90% of trials with at least one estimation incoherent change of toxicity estimates during the trial. Meanwhile, BMA-POCRM exhibits estimation incoherencies in a worst-case 0.14% of trials under scenario 10. Further, examining Fig. 4C under scenario 11, the magnitude of estimation incoherent toxicity estimates is significantly smaller for BMA-POCRM with a maximum incoherent change in toxicity estimate of 0.060 compared to POCRM for which this is 0.288. The magnitude and frequency of these incoherencies indicate that they occur as a result of numerical inaccuracies of the numerical integration scheme used by BMA-POCRM. This excludes Scenario 15 where BMA-POCRM exhibits some estimation incoherencies, however, this is the safe setting where all dose toxicity rates are below the TTR. Figure 4D shows that in trials where BMA-POCRM does exhibit estimation incoherencies, this occurs for a smaller number of cohorts. The distribution of the number of cohorts where BMA-POCRM exhibits an incoherency is consistently biased towards zero when compared to those of POCRM.

Wages and Conaway (2013) provide specific recommendations on applying the POCRM. The indifference interval skeleton parameters they recommend are used to demonstrate that estimation incoherency remains a problem even with these settings. These results can be found in Section S.2. Furthermore, we demonstrate that the BMA-POCRM either equates or improves upon the POCRM in smaller grid sizes, however, the difference in operating characteristics reduces. Simulation scenarios and additional results for smaller grid sizes are presented in Section S.4.1.

### 6.3. Comparisons to other BMA approaches

In this section, our proposed approach is compared to the other 2 BMA strategies in the literature, Zhang’s approach (Zhang et al. 2023) and Wages’ approach (Wages et al. 2011).

The difference between our proposed approach and Zhang’s approach is that, we apply BMA on the *posterior densities* of the toxicity risks $ f_{m}(R(d_{k})|\Omega_{j}) $ to first obtain an averaged (ie mixture) density $ g(R(d_{k})|\Omega_{j}) $. Then, we take the point estimate with respect to the mixture density $ \hat{R}(d_{k})=\mathbb{E}_{R(d_{k})\sim g}[R(d_{k})] $. Zhang’s approach conducted these 2 steps in the different order. They start by taking the ordering-specific point estimates of the toxicity risks $ \hat{R}_{m}(d_{k})=\mathbb{E}_{R_{m}(d_{k})\sim f_{m}}[R_{m}(d_{k})] $. Then, they apply the BMA on the point estimates to obtain the averaged toxicity risk $ R(d_{k})=\sum_{m\,=\,1}^{M}p(m|\Omega_{j})\hat{R}_{m}(d_{k}) $.

Despite the reversed order of conducting the 2 steps, Kovačević and Zhang approaches lead to the same estimates of the estimated toxicity probabilities, as stated in Lemma 6.

Theorem 6. *(Equivalence of toxicity-based BMA approaches)*. *The BMA-POCRM conducted under our proposed approach and Zhang’s approach give the same estimated toxicity probabilities.*

*Proof.* (Theorem 6). Under our proposed approach, the estimated toxicity probability

\begin{align*} \hat{R}(d_k)=&\underbrace{\int_0^1R(d_k)g(R(d_k)|\Omega_j)\,\,dR(d_k)}_{\text{our step 2}}\\ =&\int_0^1R(d_k)\underbrace{\sum_{m=1}^M p(m|\Omega_j)f_m(R(d_k)|\Omega_j)}_{\text{our step 1}}dR(d_k)\\ =&\sum_{m=1}^Mp(m|\Omega_j)\underbrace{\int_0^1R(d_k)f_m(R(d_k)|\Omega_j)dR(d_k)}_{\text{Zhang step 1}}\\ =&\underbrace{\sum_{m=1}^M p(m|\Omega_j)\hat{R}_m(d_k)}_{\text{Zhang step 2}} \end{align*}

□

The BMA strategy proposed in Wages’ approach is fundamentally different to ours and to Zhang’s. Let $ \pi_{m}(d_{k}|\Omega_{j})=p(m|\Omega_{j}) $ if $ d_{k} $ is the recommended dose under ordering $ m $, and $ \pi_{m}(d_{k}|\Omega_{j})=0 $ otherwise. Then, the combined probability of recommend dose $ d_{k} $ is $ \pi(d_{k}|\Omega_{j})=\sum_{m\,=\,1}^{M}\pi_{m}(d_{k}|\Omega_{j}) $. The dose with the largest $ \pi(d_{k}) $ will be assigned to the next cohort of patients.

Theoretically, since Zhang’s and ours approaches give exactly the same estimated toxicity probabilities, same coherency conditions in Section 5 apply to Zhang’s approach. Despite the equivalence, having the mixture posterior distribution $ g(R(d_{k})|\Omega_{j}) $ allows direct derivation of other metrics of interest, such as the 95% credible intervals around point estimates of the toxicity probability, and the probability of overdosing for each dose. Furthermore, Zhang et al. (2023) focused on fitting the BMA-POCRM on a ‘local’ subset of 5 doses around the current dose. In reality, estimates for all dose levels are important to be provided to the Dose Review Committee (as opposed to providing only the one recommended dose). On the other hand, Wages’ approach does not have a notion of the averaged toxicity probability estimates, which is the basis for the estimation coherency.

**Fig. 5.**
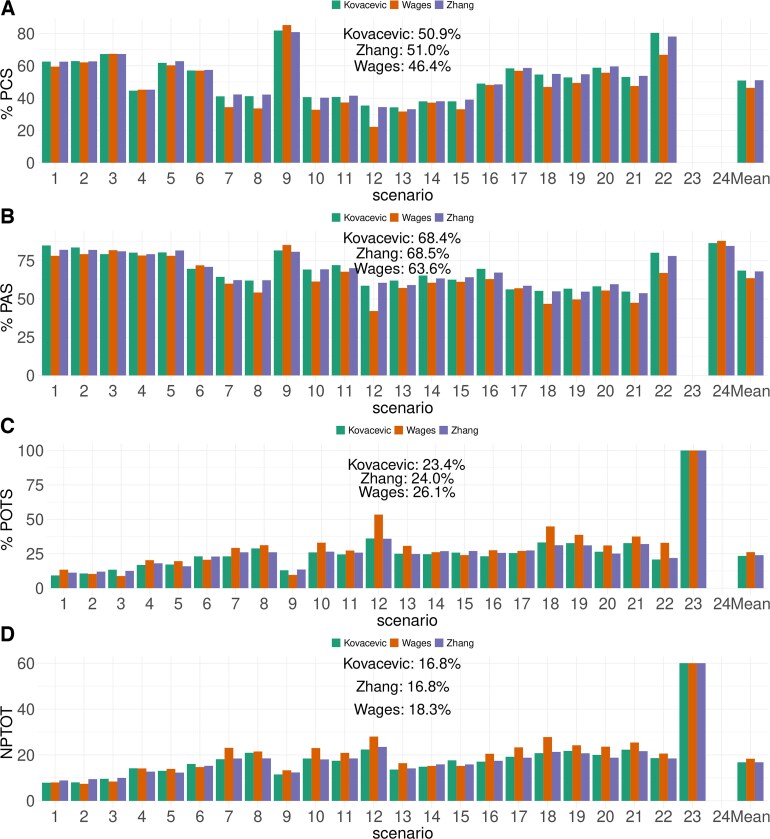
Operating characteristics under our Kovačević approach (green), Zhang’s approach (purple), and Wages’ approach (orange) under 24 scenarios. A) Proportion of correct selection. B) Proportion of acceptable selection. C) Proportion of over-toxic selection. D) Number of patients assigned to over-toxic doses. All estimations based on $ 10^{4} $ simulations.

Additional simulations are conducted under Zhang’s approach and Wages’ approach under the 24 scenarios defined in Table S1. Under Zhang’s approach, their proposed BMA strategy has been extended to the whole $ 4\times 4 $ grid to provide estimates for all doses. The same design parameters are used under all 3 approaches. Explicitly, the same 6 orderings defined in Section 6.1 is used. The toxicity skeleton is obtained via the getprior() function (Lee and Cheung 2009) with half-width $ \delta\,=\,0.02 $ and the MTC (Maximum Tolerated Combination) at $ \nu\,=\,2 $. The prior mean and variance are set to 0 and 1.34 as explained in Section 6.1. Figure 5 compares the PCS, PAS, POTS, and NPTOT between the 3 approaches. As expected, Zhang’s and ours approaches perform the same, both on average and under each scenario with all the differences being within a simulation error. Wages’ approach seems to have the lowest PCS and PAS (5% lower in both cases), and highest POTS and NPTOT (2% higher in both cases) among the 3 approaches. This is both on average across 24 scenarios and under each scenario. In particular, for the PCS in (Panel A), Wages approach has the lowest PCS under all scenarios except in scenario 9.

## 7. DISCUSSION

In this article, the concept of estimation coherency in Phase I clinical trials for drug combinations is introduced. The application of the POCRM and its operating characteristics in this combination setting are explored with the proposition of a novel method, BMA-POCRM. This approach modifies POCRM to take into account uncertainty in dose-toxicity ordering prior to making dose escalations by applying BMA. To evaluate and compare the POCRM and BMA-POCRM, a thorough study of their operating characteristics spanning model accuracy, safety and robustness to estimation incoherency are carried out. The novel method improved on its predecessor in its ability to select correct and acceptable dose levels for progression to Phase II trials. Moreover, the BMA-POCRM also led to fewer overly toxic dose recommendations. The occurrence of estimation incoherencies significantly reduced with the BMA-based method across all scenarios. For example, whilst for POCRM more than 90% of trials exhibit incoherencies for 21 of 24 scenarios, the worst-case for BMA-POCRM led to only 3.13% of trials with incoherencies. We demonstrate that these results are invariant under different dose combination grid sizes and sample sizes and are aligned with the $ 4\times 4 $ grid setting presented regardless of considered setting as seen in Section S.4.1. Furthermore, we also perform a pilot study in Section S.8 demonstrating that a run-in phase, as implemented for the 2-stage POCRM marginally reduces but does not eliminate the occurrence of estimation incoherencies.

The study of operating characteristics shows a clear improvement in performance achieved by BMA-POCRM. Combining estimates of the probability of toxicity across several candidate orderings leads to greater flexibility in predictions by the model. POCRM relies on selecting a single most probable ordering on which it bases dose allocations for the current cohort. Thus, BMA-POCRM has a distinct advantage in cases where there is weak prior knowledge of the underlying dose-toxicity orderings. In particular, the BMA component of the model allows for the specification of intermediary orderings not explicitly included in the original POCRM as shown by the improved RMSE (square-root mean squared error) of toxicity estimates across cohort sizes. Even in scenarios where the correct toxicity ordering is included as a candidate, BMA-POCRM outperforms POCRM. The BMA-POCRM thus has the potential to deliver more intuitive estimates during the conduct of the trial while demonstrating similar or better operating characteristics to the original POCRM. Moreover, irrespective of whether 1-sided or 2-sided estimation incoherencies are evaluated, the BMA-POCRM provides more coherent estimates as shown in Section S10.

Our proposed BMA method has been compared to 2 other alternative BMA methods in the literature. Zhang’s approach focuses on a local subset of doses and also applies BMA to the toxicity probabilities, which we shown theoretically is equivalent to our proposal. Wages’ approach applies BMA to the ordering-specific recommended doses and does not have averaged toxicity estimates. Theoretically, Zhang’s approach has the same coherency property as our proposed approach, whereas the notion of estimation equivalence does not apply to Wages’ approach. Simulation results confirm the equivalence of the 2 toxicity-based approach, which both have slightly better operating characteristics than Wages’ approach.

Despite the strong performance of the BMA-POCRM in the considered simulations, there are several aspects of this model that have not yet been explored. A comparison study of the impact of prior information on BMA-POCRM and POCRM performance is also necessary. This is particularly relevant when a correct ordering is included as a candidate, prior information may be available to inform these approaches, potentially leading to favourable performance for POCRM. Future work should also explore the relative performance of POCRM when restricting the model selection to only considering orderings with a posterior probability greater than a given threshold.

## Supplementary Material

kxaf035_Supplementary_Data

## Data Availability

The code for our implementations of POCRM and BMA-POCRM can be found at https://github.com/luka-kovacevic/bma-pocrm.

## References

[kxaf035-B1] Barnett H et al. 2024. A comparison of model-free phase I dose escalation designs for dual-agent combination therapies. Stat Methods Med Res. 33:203–226. 38263903 10.1177/09622802231220497 PMC10928960

[kxaf035-B2] Cheung K. 2019. *dfcrm: Dose-Finding by the Continual Reassessment Method*. R package version 0.2-2.1. https://CRAN.R-project.org/package=dfcrm Accessed on 12 Oct 2025.

[kxaf035-B3] Cheung YK. 2005. Coherence principles in dose-finding studies. Biometrika. 92:863–873.

[kxaf035-B4] Cheung YK , Chappell R. 2002. A simple technique to evaluate model sensitivity in the continual reassessment method. Biometrics. 58:671–674. 12230003 10.1111/j.0006-341x.2002.00671.x

[kxaf035-B5] Conaway MR , Dunbar S, Peddada SD. 2004. Designs for single-or multiple-agent phase I trials. Biometrics. 60:661–669. 15339288 10.1111/j.0006-341X.2004.00215.x

[kxaf035-B6] Gandhi L et al. 2014. Phase i study of neratinib in combination with temsirolimus in patients with human epidermal growth factor receptor 2–dependent and other solid tumors. J Clin Oncol. 32:68–75. 24323026 10.1200/JCO.2012.47.2787

[kxaf035-B7] Ivanova A , Kim SH. 2009. Dose finding for continuous and ordinal outcomes with a monotone objective function: a unified approach. Biometrics. 65:307–315. 18479486 10.1111/j.1541-0420.2008.01045.x PMC2819822

[kxaf035-B8] Ivanova A , Montazer-Haghighi A, Gopal Mohanty S, Durham SD. 2003. Improved up-and-down designs for phase I trials. Stat Med. 22:69–82. 12486752 10.1002/sim.1336

[kxaf035-B9] Ivanova A , Wang K. 2004. A non-parametric approach to the design and analysis of two-dimensional dose-finding trials. Stat Med. 23:1861–1870. 15195320 10.1002/sim.1796

[kxaf035-B10] Lee SM , Cheung YK. 2009. Model calibration in the continual reassessment method. Clin Trials. 6:227–238. 19528132 10.1177/1740774509105076 PMC2884971

[kxaf035-B11] Liu R et al. 2024. Design strategy and consideration for oncology dose-optimization: an industry perspective. Stat Biopharm Res. 16:338–347. 10.1080/19466315.2024.2332650

[kxaf035-B12] Mozgunov P , Jaki T. 2019. An information theoretic phase I–II design for molecularly targeted agents that does not require an assumption of monotonicity. J R Stat Soc Ser C Appl Stat. 68:347–367. 10.1111/rssc.12293 PMC6472641 31007292

[kxaf035-B13] Mozgunov P , Gasparini M, Jaki T. 2020. A surface-free design for phase I dual-agent combination trials. Stat Methods Med Res. 29:3093–3109. 32338145 10.1177/0962280220919450 PMC7612168

[kxaf035-B14] Mozgunov P et al. 2022. Practical implementation of the partial ordering continual reassessment method in a phase I combination-schedule dose-finding trial. Stat Med. 41:5789–5809. 36428217 10.1002/sim.9594 PMC10100035

[kxaf035-B15] O’Quigley J , Pepe M, Fisher L. 1990. Continual reassessment method: a practical design for phase 1 clinical trials in cancer. Biometrics. 46:33–48. 2350571

[kxaf035-B16] Park Y , Liu S. 2020. On the coherence of model-based dose-finding designs for drug combination trials. PLoS One. 15:e0242561. 33253260 10.1371/journal.pone.0242561 PMC7703981

[kxaf035-B17] Raftery AE , Madigan D, Hoeting JA. 1997. Bayesian model averaging for linear regression models. J Am Stat Assoc. 92:179–191.

[kxaf035-B18] Riviere M-K , Dubois F, Zohar S. 2015. Competing designs for drug combination in phase I dose-finding clinical trials. Stat Med. 34:1–12. 24464821 10.1002/sim.6094

[kxaf035-B19] Tighiouart M , Rogatko A. 2010. Dose finding with escalation with overdose control (ewoc) in cancer clinical trials. Statist Sci. 25:217–226.

[kxaf035-B20] Wages NA , Conaway MR. 2013. Specifications of a continual reassessment method design for phase i trials of combined drugs. Pharm Stat. 12:217–224. 23729323 10.1002/pst.1575 PMC3771354

[kxaf035-B21] Wages NA , Conaway MR, O’Quigley J. 2011. Continual reassessment method for partial ordering. Biometrics. 67:1555–1563. 21361888 10.1111/j.1541-0420.2011.01560.x PMC3141101

[kxaf035-B22] Wheeler GM. 2018. Incoherent dose-escalation in phase i trials using the escalation with overdose control approach. Stat Pap (Berl). 59:801–811. 29875549 10.1007/s00362-016-0790-7 PMC5985932

[kxaf035-B23] Yap C , Billingham LJ, Cheung YK, Craddock C, O'Quigley J. 2017. Dose transition pathways: the missing link between complex dose-finding designs and simple decision-making. Clin Cancer Res. 23:7440–7447. 28733440 10.1158/1078-0432.CCR-17-0582

[kxaf035-B24] Yin G , Yuan Y. 2009. Bayesian dose finding in oncology for drug combinations by copula regression. J R Stat Soc Ser C Appl Stat. 58:211–224.

[kxaf035-B25] Zhang J , Yan F, Wages NA, Lin R. 2023. Local continual reassessment methods for dose finding and optimization in drug-combination trials. Stat Methods Med Res. 32:2049–2063. 37593951 10.1177/09622802231192955 PMC10563380

