# Supplementary material for: Bayesian model averaging for partial ordering continual reassessment methods
Source: Biostatistics. 2025 Oct 21;26(1):kxaf035. doi: 10.1093/biostatistics/kxaf035 (PMC12538209; doi:10.1093/biostatistics/kxaf035)
Supplement: kxaf035_Supplementary_Data [file kxaf035_supplementary_data.zip › biosts-24325-File002.pdf]

Supplementary Material

S.1 SIMULATION SCENARIOS

Table S1. Scenario settings for all scenarios. Scenarios 1-12 are symmetric, meaning the MTDs are on the same diagonal. Scenarios 1A-9A have asymmetric toxicity matrices. Scenario 14 has all dose levels overly toxic. Scenario 15 has all dose levels below the TTR. Some scenarios have specific design intentions indicated here by the number of correct orderings (CO) and the number of overly toxic doses (OT). The CO number indicates the number of model choices that would have a true ordering for each corresponding setting. The OT number indicates the number of doses that have probability of toxicity greater than 110% of the TTR.

	Dose Level	Drug A											
		1	2	3	4	1	2	3	4	1	2	3	4
		**Scenario 1: 0 CO, 3 OT**				**Scenario 2: 1 CO, 3 OT**				**Scenario 3: 2 CO, 3 OT**			
	4	0.20	0.30	0.40	0.50	0.20	0.30	0.45	0.50	0.20	0.30	0.45	0.50
	3	0.10	0.20	0.30	0.45	0.16	0.20	0.30	0.40	0.10	0.20	0.30	0.40
	2	0.07	0.13	0.20	0.30	0.07	0.13	0.20	0.30	0.07	0.10	0.16	0.30
	1	0.01	0.04	0.16	0.16	0.01	0.04	0.10	0.16	0.01	0.04	0.10	0.13
		**Scenario 4: 0 CO, 6 OT**				**Scenario 5: 1 CO, 6 OT**				**Scenario 6: 2 CO, 6 OT**			
	4	0.30	0.50	0.55	0.65	0.30	0.50	0.60	0.65	0.30	0.40	0.50	0.60
	3	0.15	0.30	0.45	0.60	0.20	0.30	0.45	0.55	0.15	0.30	0.40	0.45
	2	0.05	0.20	0.20	0.40	0.07	0.15	0.30	0.40	0.10	0.15	0.30	0.40
	1	0.01	0.10	0.20	0.20	0.01	0.04	0.10	0.20	0.01	0.05	0.15	0.20
		**Scenario 7: 0 CO, 10 OT**				**Scenario 8: 1 CO, 10 OT**				**Scenario 9: 2 CO, 10 OT**			
	4	0.55	0.70	0.75	0.85	0.55	0.70	0.80	0.85	0.55	0.60	0.70	0.75
	3	0.15	0.50	0.65	0.80	0.15	0.50	0.65	0.75	0.30	0.50	0.60	0.65
	2	0.05	0.20	0.45	0.60	0.05	0.20	0.45	0.60	0.10	0.30	0.45	0.60
	1	0.01	0.10	0.30	0.40	0.01	0.10	0.30	0.40	0.10	0.05	0.30	0.40
		**Scenario 10: 0 CO, 13 OT**				**Scenario 11: 1 CO, 13 OT**				**Scenario 12: 2 CO, 13 OT**			
	4	0.70	0.85	0.90	1.00	0.70	0.85	0.95	1.00	0.60	0.65	0.75	0.80
	3	0.40	0.65	0.80	0.96	0.50	0.65	0.80	0.90	0.40	0.55	0.65	0.70
	2	0.20	0.45	0.60	0.75	0.30	0.45	0.60	0.75	0.30	0.40	0.50	0.65
**Drug B**	1	0.15	0.30	0.50	0.55	0.15	0.20	0.40	0.55	0.15	0.20	0.40	0.45
		**Scenario 1A: 0 CO**				**Scenario 2A: 1 CO**				**Scenario 3A: 2 CO**			
	4	0.30	0.40	0.50	0.55	0.30	0.40	0.47	0.60	0.30	0.40	0.50	0.55
	3	0.08	0.15	0.20	0.45	0.18	0.20	0.40	0.44	0.14	0.20	0.40	0.45
	2	0.05	0.13	0.15	0.30	0.10	0.15	0.20	0.30	0.10	0.14	0.20	0.30
	1	0.01	0.10	0.13	0.15	0.01	0.05	0.10	0.18	0.01	0.05	0.14	0.17
		**Scenario 4A: 0 CO**				**Scenario 5A: 1 CO**				**Scenario 6A: 2 CO**			
	4	0.50	0.65	0.70	0.80	0.60	0.80	0.85	0.90	0.60	0.65	0.75	0.80
	3	0.30	0.45	0.60	0.75	0.45	0.55	0.75	0.80	0.40	0.55	0.65	0.70
	2	0.05	0.20	0.40	0.55	0.30	0.40	0.55	0.75	0.30	0.40	0.50	0.65
	1	0.01	0.10	0.15	0.30	0.05	0.15	0.30	0.50	0.05	0.15	0.30	0.45
		**Scenario 7A: 0 CO**				**Scenario 8A: 1 CO**				**Scenario 9A: 2 CO**			
	4	0.60	0.70	0.75	0.85	0.55	0.70	0.75	0.80	0.55	0.60	0.75	0.80
	3	0.30	0.55	0.65	0.80	0.50	0.50	0.65	0.70	0.30	0.50	0.60	0.70
	2	0.15	0.40	0.55	0.65	0.30	0.40	0.50	0.60	0.15	0.40	0.50	0.60
	1	0.05	0.30	0.40	0.50	0.05	0.15	0.30	0.45	0.05	0.30	0.40	0.45
		**Scenario 13: 15 OT**				**Scenario 14**				**Scenario 15**			
	4	0.64	0.75	0.80	0.95	0.64	0.75	0.85	0.95	0.17	0.23	0.25	0.25
	3	0.45	0.61	0.72	0.87	0.43	0.61	0.72	0.87	0.10	0.19	0.23	0.25
	2	0.40	0.48	0.57	0.68	0.40	0.48	0.57	0.68	0.07	0.12	0.19	0.24
	1	0.30	0.40	0.50	0.57	0.37	0.42	0.50	0.57	0.05	0.07	0.17	0.19

S.2 CALIBRATION

The skeleton is optimised independently for POCRM and BMA-POCRM to ensure a fair comparison. This is done by evaluating performance under scenarios 2, 6, 5A and 13, which characterise a diverse set of scenarios. Scenario 2 and 6 have 1 and 2 correct orderings, respectively. Scenario 5A has 1 correct ordering and an

asymmetric MTD layout. Finally, scenario 13 is where all but one dose level are overly toxic. The geometric mean across simulated trials is considered as it more accurately accounts for poor performance relative to the arithmetic mean. For each scenario, 5,000 trials are simulated for which the results are aggregated by taking the geometric mean of the PCS across all 20,000 trials. Trial simulations have a total of 60 patients with 60 cohorts of size 1. The skeleton with the highest geometric mean PCS and lowest standard deviation of PCS are selected for the complete simulation study.

Two approaches for generating the underlying probability skeleton are considered. First, the indifference interval protocol defined in Lee and Cheung (2009) and implemented by the `dfcrm` `R` package (Cheung, 2019). This approach has two parameters, the prior MTD and the δ parameter, which controls the distance between the prior probability of toxicity for adjacent doses in the skeleton. Grid optimisation across these parameters is carried out to select the optimal indifference interval skeleton.

A linear skeleton is also implemented, whereby the prior probability of toxicity at the lowest dose-level is given, and the skeleton increases at a fixed interval. Recursively, this is defined as,

$$\widehat{R}^{(0)}(d_i) = \begin{cases} p_0 & \text{if } i = 1, \\ \widehat{R}^{(0)}(d_{i-1}) + \eta & \text{otherwise,} \end{cases}$$

where $\widehat{R}^{(0)}(d_i)$ is the prior probability of toxicity for dose i, p_0 is the prior probability of toxicity for the lowest dose, or *start probability* and η is the fixed increment, or *spacing*. Again, grid optimisation is carried out to find the best linear skeleton.

The calibration results for POCRM under an indifference interval are presented in Figure S1. These results show that for many prior MTDs, selecting delta as 0.03 leads to a misspecified skeleton. The same trend was empirically observed for greater values of delta. In particular, when delta is set to 0.04 a diverging marginal likelihood interval is observed for all settings of the prior MTD in at least one scenario. Since the indifference interval protocol begins at the prior MTD and obtains each adjacent skeleton value sequentially, if δ is too high, the protocol will reach the bounds of 0 and 1 quickly, resulting in several adjacent values which are arbitrarily close to 0 and 1. For example, selecting d_{15} as the prior MTD and $\delta = 0.04$ the following skeleton is obtained,

$$\{1.94e^{-12}, 4.17e^{-10}, 3.08e^{-08}, \ldots, 0.22, 0.30, 0.38\}.$$

A misspecified skeleton leads to a diverging marginal likelihood integral and is observed both for BMA-POCRM and POCRM as they share a common approach to computing the marginal likelihood in Equation (3). The skeletons proposed at this level are frequently unintuitive and result in Sall differences between dose levels at the boundaries near 0 and 1. As a result, $\delta \geq 0.03$ is excluded for the indifference interval skeleton and η (spacing) $= 0.04, 0.05, 0.06$ for the linear skeleton due to the missing values shown in Figures S1.A and S1.C, respectively.

Under the indifference interval skeleton, POCRM exhibits greatest PCS with a prior MTD d_1 and $\delta = 0.02$. Switching to a prior MTD d_2 leads to a slightly lower PCS but also lower standard deviation in performance. This is favourable as it implies greater consistency across scenarios. Assuming a linear skeleton, the design performs optimally under $p_0 = 0.4$ and $\eta = 0.03$, however, this presents significantly greater SD than when $p_0 = 0.01$ and $\eta = 0.03$. Overall, the linear skeleton is much less consistent across parametrisations when compared to the indifference interval method for the POCRM.

Similarly, Figure S2 shows that under the indifference interval for BMA-POCRM, optimal performance is observed according to mean PCS at a prior MTD d_2 and $\delta = 0.02$. This setting also leads to the Sallest SD across all valid parametrisations of the indifference interval (i.e. excluding those where $\delta > 0.03$). This setting of the indifference interval also outperforms all given parametrisations of the linear skeleton according to mean PCS. The linear skeleton with $\eta = 0.03$ and $p_0 = 0.01$ leads to lower SD, however, this comes at the expense of a point reduction in PCS.

Moving forward, a matching skeleton is selected for both POCRM and BMA-POCRM. The indifference interval skeleton with prior MTD d_2 and $\delta = 0.02$ is used due to the balance between aggregate performance and consistency across both the methods included (i.e. comparatively low SD).

Our simulation study includes the indifference interval skeleton settings recommended by Wages and Conaway (2013) (prior MTD: d_4; $\delta = 0.06$). In addition, we simulated 1,000 trials under the POCRM with these settings observed a PCS of 57.2%, PAS of 84.6%, POTS of 6.6%, and NPTOT of 5.362 per trial, which underperforms the POCRM under our settings. This setting yields at least one estimation in 94.5% of simulated trials, and an average of 4.174 incoherencies per trial.

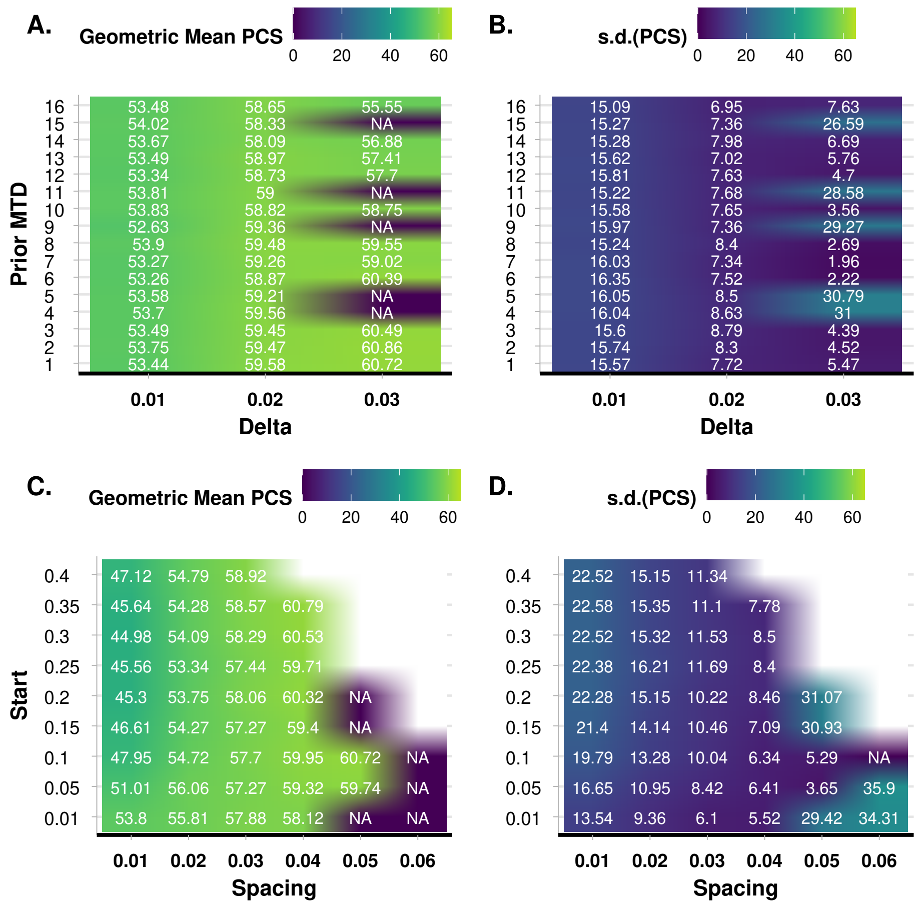

Fig. S1. Results for prior calibration of skeleton parameters for the POCRM with model selection. Panel A. Geometric mean of PCS for various values of the indifference interval skeleton under the POCRM model. Panel B. Standard deviation of PCS across scenarios selected for calibration when applying the indifference interval skeleton under the POCRM model. Panel C. Geometric mean of PCS for various values of the linear skeleton under the POCRM model. Panel D. Standard deviation of PCS across scenarios selected for calibration when applying the linear skeleton under the POCRM model. Missing values indicate at least one diverging marginal likelihood integral over simulations.

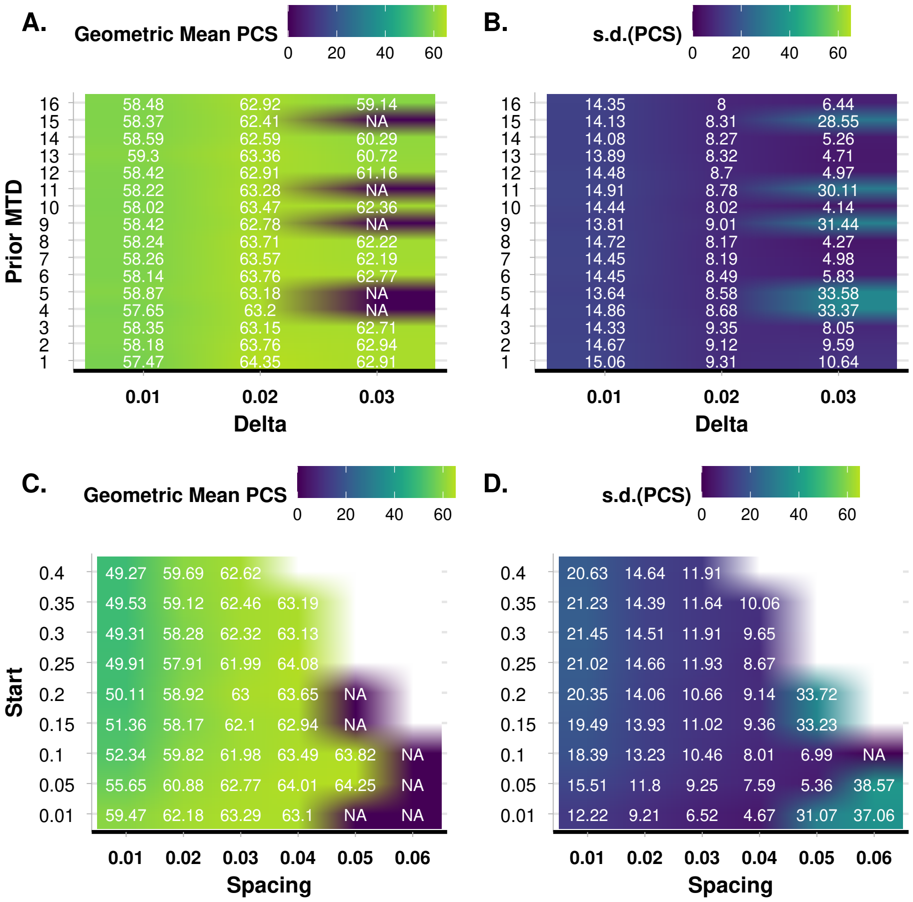

Fig. S2. Results for prior calibration of skeleton parameters for the BMA-POCRM. Panel A. Geometric mean of PCS for various values of the indifference interval skeleton under the BMA-POCRM model. Panel B. Standard deviation of PCS across scenarios selected for calibration when applying the indifference interval skeleton under the BMA-POCRM model. Panel C. Geometric mean of PCS for various values of the linear skeleton under the BMA-POCRM model. Panel D. Standard deviation of PCS across scenarios selected for calibration when applying the linear skeleton under the BMA-POCRM model. Missing values indicate at least one diverging marginal likelihood integral over simulations.

S.3 VARIANCE CALIBRATION

The effect of altering the variance of the prior distribution of the a parameter of the working model is also explored in Supplementary Materials. It was found that the prior variance does not have any influence on the mean PCS for neither of the two approaches, and hence the value of 1.34 is used for further simulations.

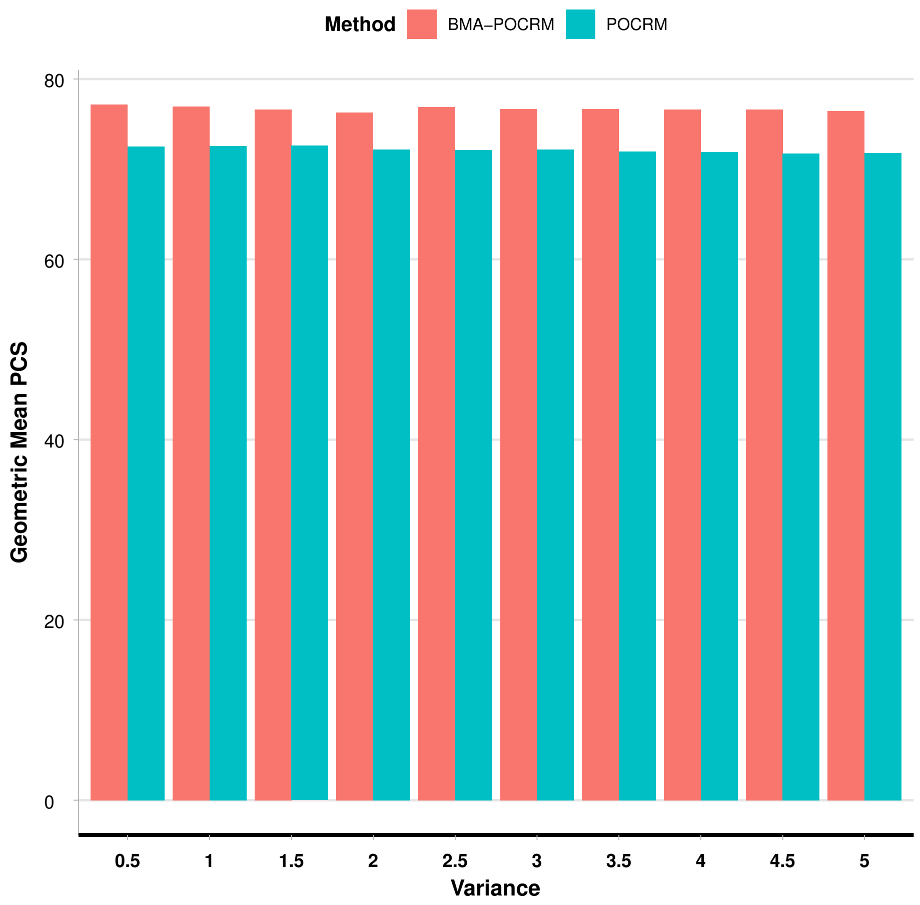

Fig. S3. Geometric mean of PCS across all scenarios. The optimal skeleton, $\mathcal{S}$, is used here.

S.4 FURTHER SIMULATION RESULTS

The distribution of changes following updates to the posterior model probabilities are given in Figure S4. Generally, the changes observed in model probabilities are similar across the POCRM and BMA-POCRM models.

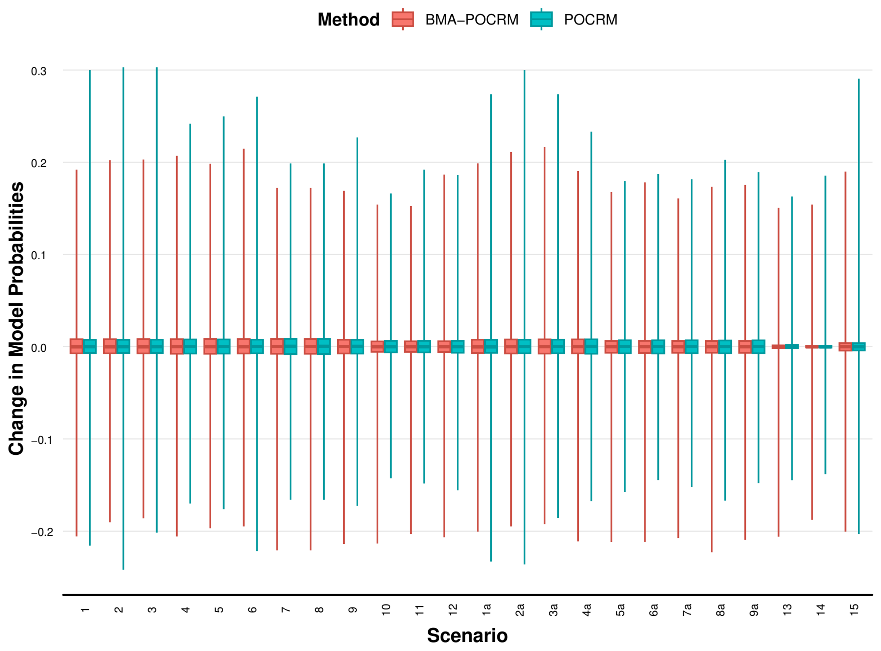

Fig. S4. Distribution of changes in posterior model probabilities following the induction of each cohort for simulated trials with cohort size 1.

S.4.1 Additional Grid Sizes

In addition to the simulation scenarios in Section S.1, we also conduct simulation studies for grid sizes of 2×3, 3×3, and 4×4 for population sizes of 30, 45, and 60.

Table S2. Scenario settings for 3×3 grid simulations. The highlighted dose levels indicate the true MTDs.

Dose Level		Drug A					
		1	2	3	1	2	3
		**Scenario 1**			**Scenario 2**		
Drug B	3	0.10	0.20	0.30	0.20	0.30	0.50
	2	0.07	0.13	0.25	0.13	0.20	0.30
	1	0.01	0.04	0.16	0.04	0.10	0.16
		**Scenario 3**			**Scenario 4**		
	3	0.18	0.37	0.56	0.42	0.68	0.93
	2	0.12	0.30	0.49	0.30	0.53	0.84
	1	0.07	0.20	0.43	0.18	0.39	0.75

Table S3. Scenario settings for 3×3 grid simulations. The highlighted dose levels indicate the true MTDs.

Dose Level		Drug A					
		1	2	3	1	2	3
		**Scenario 1**			**Scenario 2**		
Drug B	3	0.05	0.15	0.30	0.15	0.30	0.45
	2	0.01	0.06	0.20	0.05	0.20	0.30
		**Scenario 3**			**Scenario 4**		
	3	0.15	0.30	0.60	0.50	0.80	0.90
	2	0.05	0.15	0.50	0.30	0.60	0.70

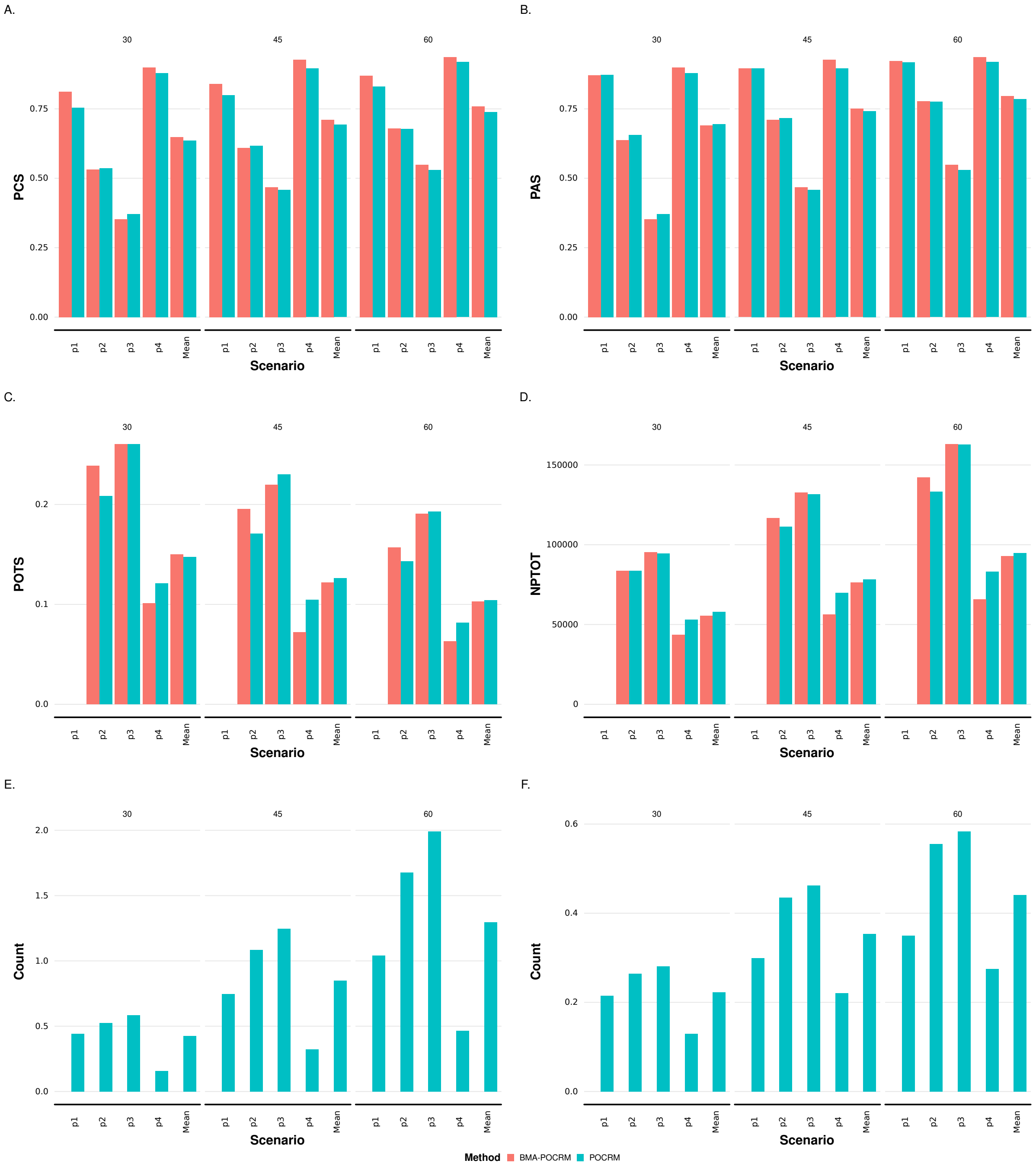

Fig. S5. Operating characteristics and incoherency rates for the 2×3 grid simulations.

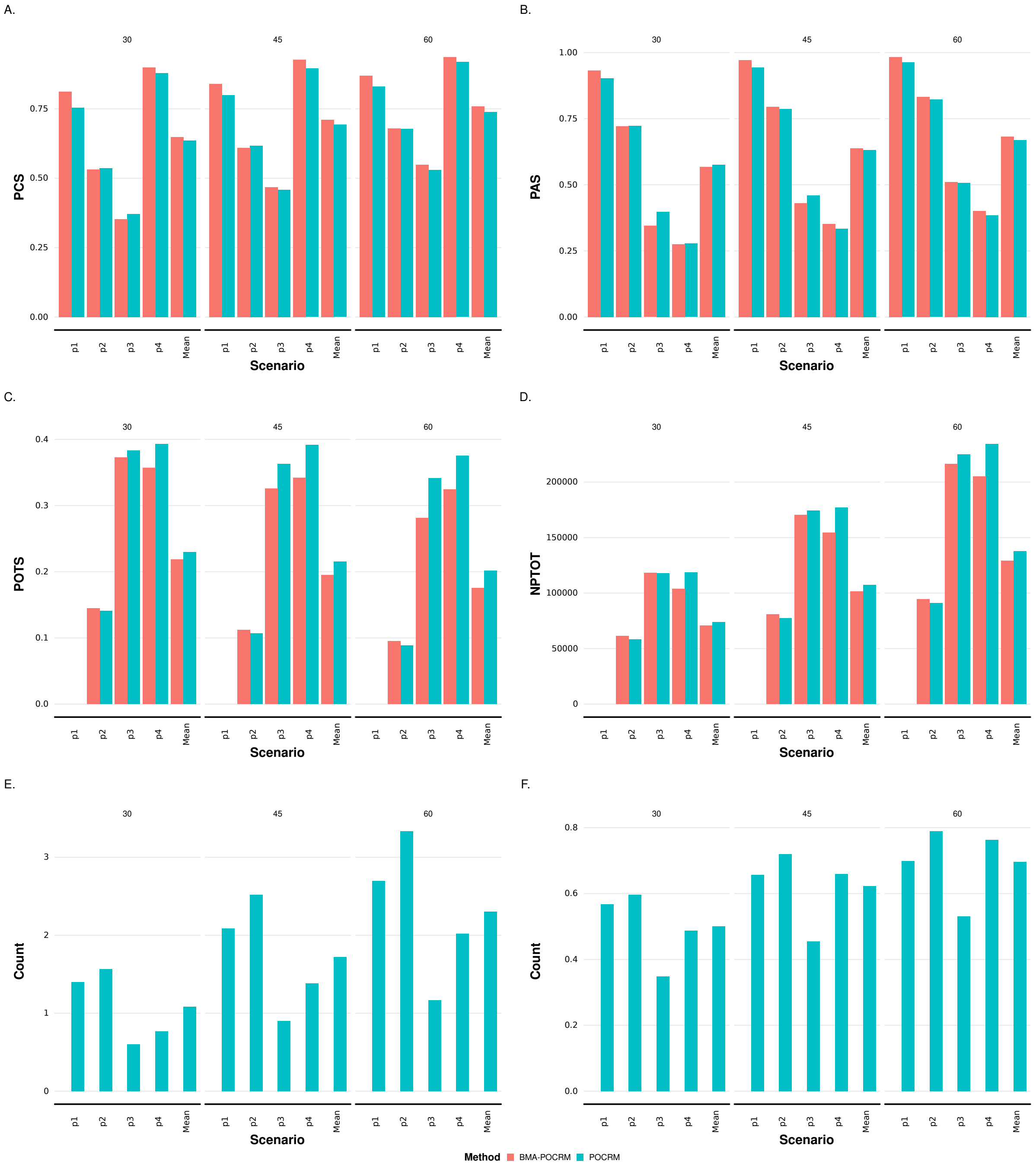

Fig. S6. Operating characteristics and incoherency rates for the 3×3 grid simulations.

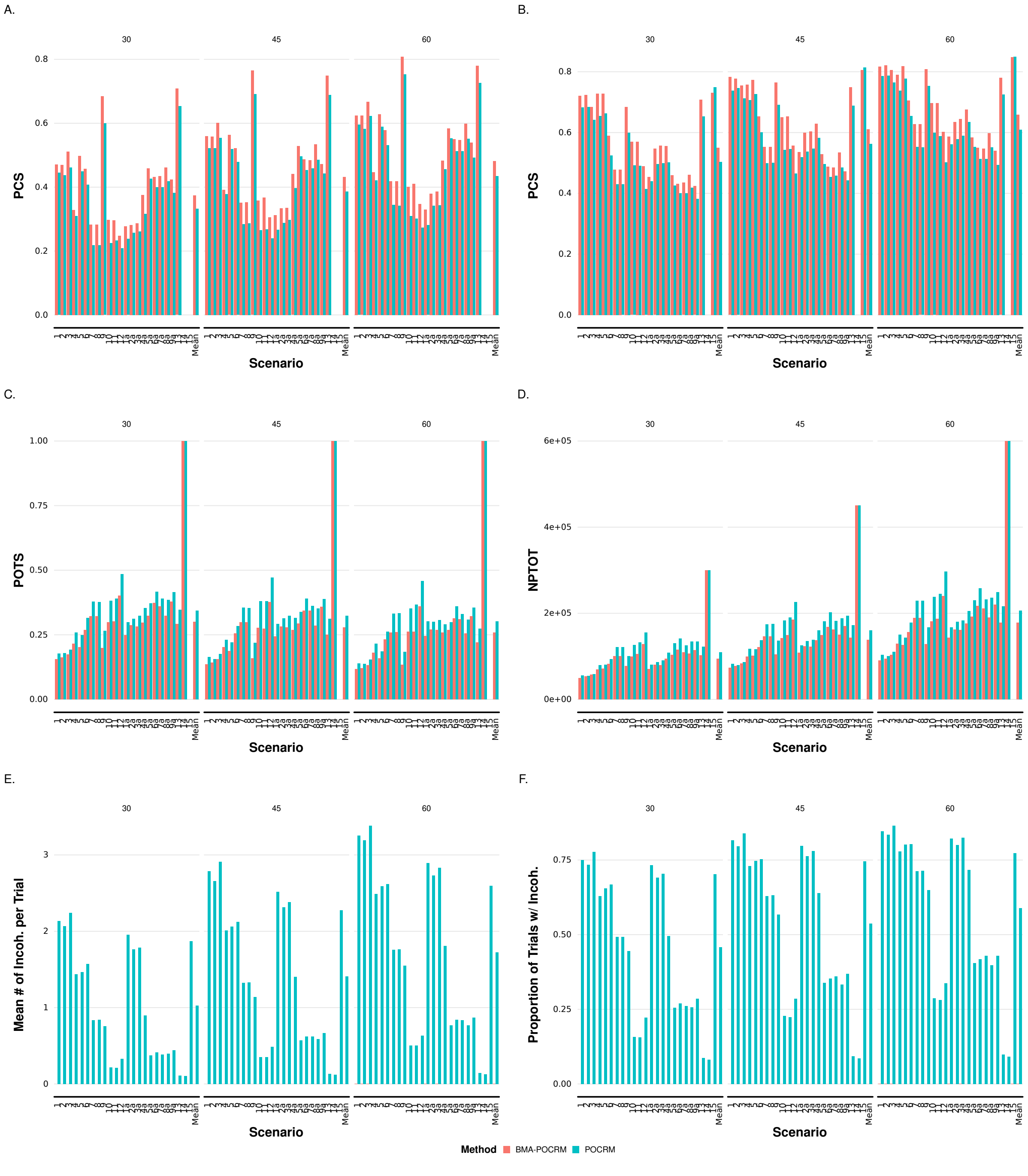

Fig. S7. Operating characteristics and incoherency rates for the 4×4 grid simulations.

S.5 TRIAL DATA

Table S4. Real trial data generated by Gandhi et al. (2014) where each cell represents y_j/n_j the number of observed DLTs and the number of patients assigned to each combination.

		Temsirolimus (mg)			
		15	*25*	*50*	*75*
Neratinib (mg)	*240*	2/4	–	–	–
	200	0/4	1/8	1/2	–
	160	1/4	1/4	0/5	3/6
	120	0/2	0/4	1/5	0/4

Table S5. Key for doses allocated in the Gandhi et al. (2014) trial.

		Temsirolimus (mg)			
		15	*25*	*50*	*75*
Neratinib (mg)	*240*	d_{12}	–	–	–
	200	d_9	d_{10}	d_{11}	–
	160	d_5	d_6	d_7	d_8
	120	d_1	d_2	d_3	d_4

Table S6. Dose allocations with POCRM in the motivating trial (Gandhi et al., 2014) where each cell represents y_j/n_j the number of observed DLTs and the number of patients assigned to each combination. The starting combination is d_5.

		Temsirolimus (mg)			
		15	*25*	*50*	*75*
Neratinib (mg)	*240*	15/30	–	–	–
	200	0/1	1/16	0/0	–
	160	0/1	0/0	0/1	1/1
	120	0/0	0/0	0/1	0/2

Table S7. Dose allocations with BMA-POCRM in the motivating trial (Gandhi et al., 2014) where each cell represents y_j/n_j the number of observed DLTs and the number of patients assigned to each combination. The starting combination is d_5.

		Temsirolimus (mg)			
		15	*25*	*50*	*75*
Neratinib (mg)	*240*	13/28	–	–	–
	200	0/1	1/17	1/2	–
	160	0/1	0/0	0/0	1/1
	120	0/0	0/0	0/0	0/2

S.6 PROOFS of THEOREMS

Without loss of generality, the proofs below consider the case where the estimated DLT probabilities increase after observing a non-DLT. The other case, i.e. the estimated DLT probability decreases after observing a DLT, is symmetric. For 2×2 combinations, there are only 4 possible cases where an incoherence w.r.t. estimation can occur.

($\mathbf{C_1}$) A non-DLT is observed at d_1, and the selected ordering changes from $\mathcal{O}_1$ to $\mathcal{O}_2$. The estimated DLT probability at d_2 increases.

($\mathbf{C_2}$) A non-DLT is observed at d_1, and the selected ordering changes from $\mathcal{O}_2$ to $\mathcal{O}_1$. The estimated DLT probability at d_3 increases.

($\mathbf{C_3}$) A non-DLT is observed at d_4, and the selected ordering changes from $\mathcal{O}_1$ to $\mathcal{O}_2$. The estimated DLT probability at d_2 increases.

($\mathbf{C_4}$) A non-DLT is observed at d_4, and the selected ordering changes from $\mathcal{O}_2$ to $\mathcal{O}_1$. The estimated DLT probability at d_3 increases.

Theorem 6 show that our proposed approach and Zhang's approach give exactly the same estimated toxicity probabilities, and thus all the other proofs in this section follows the Zhang description of the BMA-POCRM for the ease of derivations.

Proof (Lemma 1)
Under case (C_1), after a non-DLT has been observed at d_1, if the selected ordering remains to be $\mathcal{O}_1$, the CRM coherence ensures that $\hat{p}^l_{1,1} \geq \hat{p}^{l+1}_1$, and thus $\hat{a}^{(l+1)}_1 \geq \hat{a}^{(l)}_1$. As a result, $\hat{p}^{l+1}_2 = \alpha_2^{\hat{a}_1^{(l+1)}} \leq \alpha_2^{\hat{a}_1^{(l)}} = \hat{p}^l_2$, the POCRM is coherent w.r.t. estimation. The other three case follows under the same reasoning. □

Proof (Theorem 2)
Let $\hat{p}^l_k$ be estimated DLT probability of d_k after the first $l \in \mathbb{N}$ patients. Under case (C_1), the coherence of the POCRM requires

$$\alpha_2^{\hat{a}_1^{(l)}} = p^l_2 \geq p^{l+1}_2 = \alpha_3^{\hat{a}_2^{(l+1)}}.$$

Although the value of the estimated parameter values are unknown without simulation, **?** shown that $\hat{a}^{(l)}_1 \in [\min \mathcal{A}_1, \max \mathcal{A}_1]$, $\hat{a}^{(l)}_2 \in [\min \mathcal{A}_2, \max \mathcal{A}_2]$, where $\mathcal{A}_1 = \{\log R_1/\log\alpha_1,\ \log R_2/\log\alpha_2,\ \log R_3/\log\alpha_3,\ \log R_4/\log\alpha_4\}$, $\mathcal{A}_2 = \{\log R_1/\log\alpha_1,\ \log R_2/\log\alpha_3,\ \log R_3/\log\alpha_2,\ \log R_4/\log\alpha_4\}$. Hence, a sufficient condition would be to require the upper bound of the LHS to be smaller than the lower bound of the RHS, which gives

$$\alpha_2^{\hat{a}_1^{\min}} \leq \alpha_3^{\hat{a}_2^{\max}},$$

where $\tilde{a}^{(1)}_{\min} = \min \mathcal{A}_1$, $\tilde{a}^{(1)}_{\max} = \max \mathcal{A}_1$, $\tilde{a}^{(2)}_{\min} = \min \mathcal{A}_2$, $\tilde{a}^{(2)}_{\max} = \max \mathcal{A}_2$.

Similarly, under case (C_2), the POCRM coherence requires

$$\alpha_2^{\hat{a}_2^{(l)}} = p^l_3 \geq p^{l+1}_3 = \alpha_3^{\hat{a}_1^{(l+1)}}.$$

A sufficient condition would be

$$\alpha_2^{\hat{a}_2^{\min}} \leq \alpha_3^{\hat{a}_1^{\max}}.$$

The case ($C3$) requires the same condition as ($C1$), and ($C4$) is the same as (C_2). □

Lemma 7 and Lemma 8 provide upper bounds on the changes of the posterior ordering probability and estimates of model parameter after enrolling one more patient, respectively, under case (C_1). This will be used in the proof Theorem 3.

Lemma 7 (Upper bound on the posterior ordering probability) *When the selected ordering changes from $\mathcal{O}_1$ to $\mathcal{O}_2$ from the lth to the $(l+1)$th patient after observing a non-DLT at d_1, let $\Delta = p(1|\Omega_l) - p(1|\Omega_{l+1})$, $l = 1, 2, \ldots$, be the change in the posterior probabilities of ordering 1, then*

$$|\Delta| \leq \frac{1}{1 - \log \alpha_1}. \tag{14}$$

Lemma 8 (Upper bound on the posterior estimates of the model parameter) *When the selected ordering changes from $\mathcal{O}_1$ to $\mathcal{O}_2$ from the lth to the $(l+1)$th patient after observing a non-DLT at d_1, let $\Delta_a = \hat{a}_2^{(l+1)} - \hat{a}_2^{(l)}$, $l = 1, 2, \ldots$, be the change in the posterior estimates of the model parameter under ordering 2, then*

$$\Delta_a \leq \frac{1 - 2\log \alpha_1}{1 - \log \alpha_1} \hat{a}_2^{\max} - \frac{1}{(1 - \log \alpha_1)^2}, \tag{15}$$

where $\hat{a}_2^{\max} = \max\{\log R_1 / \log \alpha_1,\ \log R_2 / \log \alpha_3,\ \log R_3 / \log \alpha_2,\ \log R_4 / \log \alpha_4\}$.

Proof (Lemma 7) Let $\mathcal{L}^{(m)}(a; \Omega_l)$ be the likelihood under ordering m given the first l patients.

$$\mathcal{L}^{(m)}(a; \Omega_l) = \prod_{k=1}^{l} \psi_m(x_k; a)^{y_k} [1 - \psi_m(x_k; a)^{1-y_k}],$$

where x_k, y_k refer to the combination assigned to the kth patients and the binary DLT outcome of the kth patient. To simplify the notations, let

$$\int \mathcal{L}^{(m)}(a; \Omega_l) \pi(a) da = L_l^{(m)}, \qquad m = 1, 2,$$

where $\pi(a)$ denotes the prior distribution of a, which is assumed Exp(1), and let $S_l = \sum_m L_l^{(m)}$. Then, the posterior ordering probability is defined as $p(1|\Omega_l) = \frac{L_l^{(1)}}{S_l}$ under equal prior ordering probabilities $p(m) = 1/2$. When the $(li + 1)$th patient is enrolled to d_1 and results in a non-DLT,

$$L_{l+1}^{(m)} = \int (1 - \alpha_1^a) \mathcal{L}^m(a; \Omega_l) \pi(a) da = L_l^{(m)} - \int \alpha_1^a \mathcal{L}^m(a; \Omega_l) \pi(a) da.$$

Hence,

$$\begin{aligned}
|L_l^{(m)} - L_{l+1}^{(m)}| &= \left| L_l^{(m)} - L_l^{(m)} + \int \alpha_1^a \mathcal{L}^{(m)}(a; \Omega_l) \pi(a) da \right| \\
&= \left| \int \alpha_1^a \mathcal{L}^{(m)}(a; \Omega_l) \pi(a) da \right| \\
&\leq \int \alpha_1^a \mathcal{L}^{(m)}(a; \Omega_l) \pi(a) da,
\end{aligned}$$

by triangle inequality. Note that this upper bound is maximised when $l = 0$, in which case, the integral gives $1/(1 - \log \alpha_1)$. Hence,

$$\begin{aligned}
p(1|\Omega_{l+1}) =& \frac{L_{l+1}^{(1)}}{L_{l+1}^{(1)} + L_{i+1}^{(2)}} \geq \frac{L_l^{(1)} - \frac{1}{1-\log \alpha_1}}{S_l + \frac{2}{1-\log \alpha_1}} \\
=& \frac{p(1|\Omega_l) S_l - \frac{1}{1-\log \alpha_1}}{S_l + \frac{2}{1-\log \alpha_1}} \\
=& \frac{p(1|\Omega_l)}{1 + \frac{2}{S_l(1-\log \alpha_1)}} - \frac{1}{2 + S_l(1 - \log \alpha_1)} \\
\geq& \frac{p(1|\Omega_l)}{1 + \frac{1}{1-\log \alpha_1}} - \frac{1}{2 + 2(1 - \log \alpha_1)},
\end{aligned}$$

because the lower bound is minimised when S_l is minimised, the derivative w.r.t. $S_l > 0$. This happens when $l = 0$ and $S_0 = 2$. Since ordering 2 is selected at $l+1$, $p(1|\Omega_{l+1}) \leq 1/2$, which gives

$$\frac{p(1)}{1+\frac{1}{1-\log\alpha_1}} - \frac{1}{2+2(1-\log\alpha_1)} \leq \frac{1}{2} \qquad \Rightarrow \qquad p(1|\Omega_l) \leq \frac{3-\log\alpha_1}{2-2\log\alpha_1}.$$

The change in posterior ordering probabilities

$$\begin{aligned}|\Delta| =& |p(1|\Omega_{l+1}) - p(1|\Omega_l)| \\ \leq& \left| p(1|\Omega_{l+1}) - \frac{p(1|\Omega_l)}{1+\frac{1}{1-\log\alpha_1}} + \frac{1}{2+2(1-\log\alpha_1)} \right| \\ =& \left| \left(p(1|\Omega_l) + \frac{1}{2}\right) \frac{1}{2-\log\alpha_1} \right| \\ \leq& \left| \left(\frac{3-\log\alpha_1}{2-2\log\alpha_1} + \frac{1}{2}\right) \frac{1}{2-\log\alpha_1} \right| \\ =& \left| \frac{1}{1-\log\alpha_1} \right| = \frac{1}{1-\log\alpha_1}.\end{aligned}$$

□

Proof (Theorem 3)
The case (C_1) is assumed to start with. Let $\tilde{p}_2^{(l)}$ be the estimated DLT probability for d_2 after the lth patient under the BMA-POCRM. Then,

$$\begin{aligned}\tilde{p}_2^{(l)} =& \alpha_2^{\hat{a}_l^{(1)}} p(1|\Omega_l) + \alpha_3^{\hat{a}_2^{(l)}} [1 - p(1|\Omega_l)] \\ \tilde{p}_2^{(l+1)} =& \alpha_2^{\hat{a}_1^{(l+1)}} [p(1|\Omega_l) - \Delta] + \alpha_3^{\hat{a}_2^{(l+1)}} [1 - p(1|\Omega_l) + \Delta]\end{aligned}$$

Estimation-coherency requires $\tilde{p}_2^{(i)} \geq \tilde{p}_2^{(i+1)}$, which leads to

$$\left(\alpha_3^{\hat{a}_2^{(l+1)}} - \alpha_2^{\hat{a}_1^{(l+1)}}\right) \Delta \leq \left(\alpha_2^{\hat{a}_1^{(l)}} - \alpha_2^{\hat{a}_1^{(l+1)}}\right) p(1|\Omega_l) + \left(\alpha_3^{\hat{a}_2^{(l)}} - \alpha_3^{\hat{a}_2^{(l+1)}}\right) [1 - p(1|\Omega_l)] \tag{16}$$

The RHS of (16) is always non-negative. Hence, if $\left(\alpha_3^{\hat{a}_2^{(l+1)}} - \alpha_2^{\hat{a}_1^{(l+1)}}\right) \Delta < 0$, the inequality is always valid. If $\left(\alpha_3^{\hat{a}_2^{(l+1)}} - \alpha_2^{\hat{a}_1^{(l+1)}}\right) \Delta \geq 0$, (16) is equivalent to

$$|\Delta| \leq p(1|\Omega_l) + \frac{\alpha_3^{\hat{a}_2^{(l)}} - \alpha_3^{\hat{a}_2^{(l+1)}}}{\left|\alpha_3^{\hat{a}_2^{(l+1)}} - \alpha_2^{\hat{a}_1^{(l+1)}}\right|} + \frac{\alpha_2^{\hat{a}_1^{(l)}} - \alpha_3^{\hat{a}_2^{(l)}}}{\left|\alpha_3^{\hat{a}_2^{(l+1)}} - \alpha_2^{\hat{a}_1^{(l+1)}}\right|} p(1|\Omega_l) \tag{17}$$

Note that the 2nd term of (17) is always positive by the CRM-coherency.

- When $\alpha_2^{\hat{a}_1^{(l)}} \geq \alpha_3^{\hat{a}_2^{(l)}}$, the 3rd term of (17) is also positive, and it's sufficient to have $|\Delta| < p(1|\Omega_i)$, which is true by construction.
- When $\alpha_2^{\hat{a}_1^{(l)}} < \alpha_3^{\hat{a}_2^{(l)}}$,

$$\text{Equation (17)} \quad \Leftrightarrow \quad |\Delta| \leq p(1|\Omega_l) + \frac{\alpha_3^{\hat{a}_2^{(l)}} - \alpha_3^{\hat{a}_2^{(l+1)}}}{\left|\alpha_3^{\hat{a}_2^{(l+1)}} - \alpha_2^{\hat{a}_1^{(l+1)}}\right|} - \frac{\alpha_3^{\hat{a}_2^{(l)}} - \alpha_2^{\hat{a}_1^{(l)}}}{\left|\alpha_3^{\hat{a}_2^{(l+1)}} - \alpha_2^{\hat{a}_1^{(l+1)}}\right|} p(1|\Omega_l). \tag{18}$$

–If $\alpha_2^{\tilde{a}_l^{(1)}} \geq \alpha_3^{\hat{a}_2^{(l+1)}}$, i.e. the POCRM is estimation-coherent after a change of the selected ordering, see the proof for Theorem 4.

–If $\alpha_2^{\tilde{a}_l^{(1)}} < \alpha_3^{\hat{a}_2^{(l+1)}}$,

$$\text{Equation (18)} \quad \Leftrightarrow \quad |\Delta| \leq \left(1 - \frac{\alpha_3^{\hat{a}_2^{(l)}} - \alpha_2^{\hat{a}_1^{(l)}}}{\alpha_3^{\hat{a}_2^{(l+1)}} - \alpha_2^{\hat{a}_1^{(l+1)}}}\right) p(1|\Omega_l) + \frac{\alpha_3^{\hat{a}_2^{(l)}} - \alpha_3^{\hat{a}_2^{(l+1)}}}{\alpha_3^{\hat{a}_2^{(l+1)}} - \alpha_2^{\hat{a}_1^{(l+1)}}}. \tag{19}$$

A sufficient condition for Equation (19) is

$$|\Delta| \leq \frac{\alpha_3^{\hat{a}_2^{(l)}} - \alpha_3^{\hat{a}_2^{(l+1)}}}{\alpha_3^{\hat{a}_2^{(l+1)}} - \alpha_2^{\hat{a}_1^{(l+1)}}}.$$

From Lemma 7, a general upper bound for $|\Delta|$ is $1/(1 - \log \alpha_1)$, and thus it suffices to have

$$\frac{1}{1 - \log \alpha_1} \leq \frac{\alpha_3^{\hat{a}_2^{(l)}} - \alpha_3^{\hat{a}_2^{(l+1)}}}{\alpha_3^{\hat{a}_2^{(l+1)}} - \alpha_2^{\hat{a}_1^{(l+1)}}}. \tag{20}$$

However, the above condition cannot be checked without simulation in practice, and we derive an easy to check condition below. The RHS of (20) can be written as

$$\frac{\alpha_3^{\hat{a}_2^{(l+1)}} \left(\alpha_3^{\tilde{a}_l^{(2)} - \hat{a}_2^{(l+1)}} - 1\right)}{1 - \alpha_2^{\hat{a}_1^{(l+1)}} / \alpha_3^{\hat{a}_2^{(l+1)}}} \geq \frac{\alpha_3^{\tilde{a}_{\min}^{(2)}} \left(\alpha_3^U - 1\right)}{1 - \alpha_2^{\tilde{a}_{\min}^{(1)}} / \alpha_3^{\tilde{a}_{\max}^{(2)}}},$$

where U is an upper bound of the change in the parameter estimates, which is provided by Lemma 8. Hence, the sufficient condition under case (C_1) is

$$\frac{1}{1 - \log \alpha_1} \leq \frac{\alpha_3^{\tilde{a}_{\min}^{(2)}} \left(\alpha_3^{U_1} - 1\right)}{1 - \alpha_2^{\tilde{a}_{\min}^{(1)}} / \alpha_3^{\tilde{a}_{\max}^{(2)}}}, \qquad \text{where } U_1 = \frac{1 - 2\log \alpha_1}{1 - \log \alpha_1} \hat{a}_2^{\max} - \frac{1}{(1 - \log \alpha_1)^2}. \tag{21}$$

Under case (C_2), the roles of ordering 1 and 2 has been changed, and thus exchange $\tilde{a}^{(1)}$ and $\tilde{a}^{(2)}$ in Equation (21) gives a sufficient condition for this case. Under case (C_3), the only change in condition (21) is to change all the α_1's to α_4. Finally, the case (C_4) requires the same condition as (C_2) after changing all the α_1's to α_4. □

Proof (Theorem 4)
Under case (C_1), when the POCRM is coherent,

$$\begin{aligned}
\alpha_2^{\hat{a}_1^{(l)}} \geq \alpha_3^{\hat{a}_2^{(l+1)}} \quad & \Leftrightarrow \quad -\alpha_3^{\hat{a}_2^{(l+1)}} \geq -\alpha_2^{\hat{a}_1^{(l)}} \\
& \Leftrightarrow \quad \alpha_3^{\hat{a}_2^{(l)}} - \alpha_3^{\hat{a}_2^{(l+1)}} \geq \alpha_3^{\hat{a}_2^{(l)}} - \alpha_2^{\hat{a}_1^{(l)}} \\
& \Rightarrow \quad \alpha_3^{\hat{a}_2^{(l)}} - \alpha_3^{\hat{a}_2^{(l+1)}} \geq \left(\alpha_3^{\hat{a}_2^{(l)}} - \alpha_2^{\hat{a}_1^{(l)}}\right) p(1|\Omega_l) \\
& \Leftrightarrow \quad \frac{\alpha_3^{\hat{a}_2^{(l)}} - \alpha_3^{\hat{a}_2^{(l+1)}}}{\left|\alpha_3^{\hat{a}_2^{(l+1)}} - \alpha_2^{\hat{a}_1^{(l+1)}}\right|} - \frac{\alpha_3^{\hat{a}_2^{(l)}} - \alpha_2^{\hat{a}_1^{(l)}}}{\left|\alpha_3^{\hat{a}_2^{(l+1)}} - \alpha_2^{\hat{a}_1^{(l+1)}}\right|} p(1|\Omega_l) \geq 0.
\end{aligned}$$

Hence,

$$|\Delta| \leq p(1|\Omega_l) \leq p(1|\Omega_l) + \frac{\alpha_3^{\hat{a}_2^{(l)}} - \alpha_3^{\hat{a}_2^{(l+1)}}}{\left|\alpha_3^{\hat{a}_2^{(l+1)}} - \alpha_2^{\hat{a}_1^{(l+1)}}\right|} - \frac{\alpha_3^{\hat{a}_2^{(l)}} - \alpha_2^{\hat{a}_1^{(l)}}}{\left|\alpha_3^{\hat{a}_2^{(l+1)}} - \alpha_2^{\hat{a}_1^{(l+1)}}\right|} p(1|\Omega_l),$$

condition (17) is valid. Hence, the BMA-POCRM is coherent. The other 3 cases are all similar. □

Proof (Theorem 5)
Consider case (C_1) to start with. When both the POCRM and the BMA-POCRM are incoherent, the magnitudes of the incoherences are

$$\begin{aligned} M_S =& \alpha_3^{\hat{a}_2^{(l+1)}} - \alpha_2^{\tilde{a}_l^{(1)}} \\ M_A =& \alpha_2^{\hat{a}_1^{(l+1)}}[p(1|\Omega_l) - \Delta] + \alpha_3^{\hat{a}_2^{(l+1)}}[1 + \Delta - p(1|\Omega_l)] - \alpha_2^{\tilde{a}_l^{(1)}} p(1|\Omega_l) - \alpha_3^{\tilde{a}_l^{(2)}}[1 - p(1|\Omega_l)] \\ =& \Delta\left(\alpha_3^{\hat{a}_2^{(l+1)}} - \alpha_2^{\tilde{a}_l^{(1)}}\right) - \left(\alpha_2^{\hat{a}_1^{(l+1)}} - \alpha_2^{\hat{a}_1^{(l+1)}}\right)[p(1|\Omega_l) - \Delta] - \left(\alpha_3^{\hat{a}_2^{(l)}} - \alpha_3^{\hat{a}_2^{(l+1)}}\right)[1 - p(1|\Omega_l)]. \end{aligned}$$

The last two terms are both positive, and thus

$$M_s \le \Delta M_A \le \frac{1}{1 - \log \alpha_1} M_S,$$

based on Lemma 7.

Case (C_2) is the same as case (C_1), and cases (C_3) and (C_4) both give

$$M_A \le \frac{1}{1 - \log \alpha_4} M_S.$$

Hence, under all possible cases,

$$M_A \le \max\left\{\frac{1}{1 - \log \alpha_1}, \frac{1}{1 - \log \alpha_4}\right\} M_S = \frac{1}{1 - \log \alpha_4} M_S.$$

□

S.7 CHECK THE SUFFICIENT COHERENCE CONDITIONS

The sufficient conditions for the estimation coherency are checked in Figure S8 under four scenarios of true toxicity probabilities. There is no skeleton that can satisfy the sufficient conditions for the model selection version of the POCRM. Orange area corresponds to values of (α_2, α_3) that satisfy the sufficient condition for the BMA-POCRM, which is not restrictive.

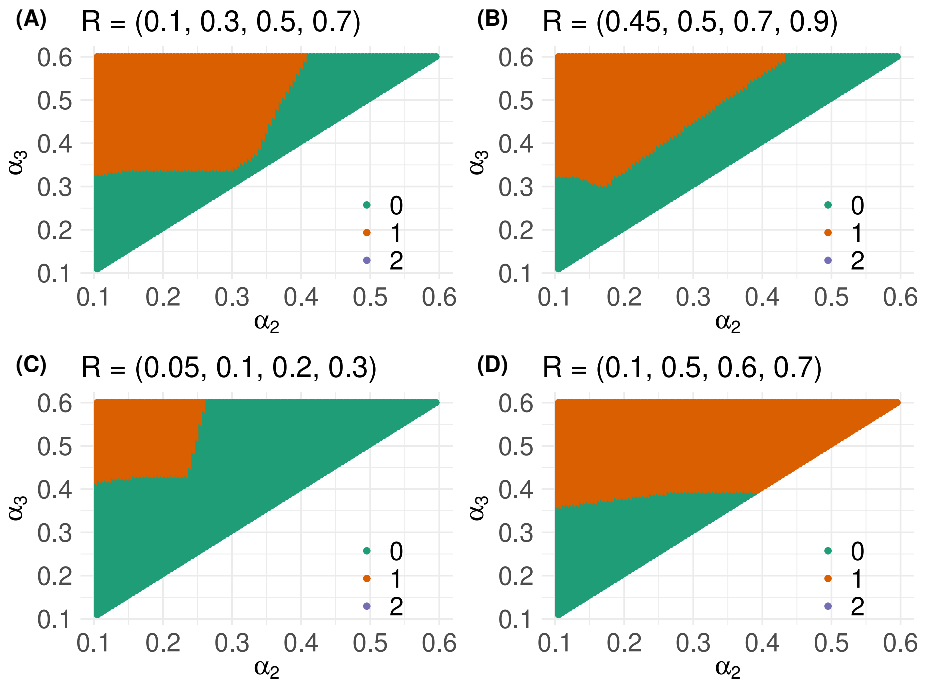

Fig. S8. Values of (α_2, α_3) satisfying the sufficient conditions for the coherency of the POCRM and BMA-POCRM under four scenarios. α_1 and α_4 fixed at 0.1 and 0.6. Colours correspond to the number of coherent designs, neither design coherent (green), only the BMA-POCRM coherence (orange), both designs coherent (purple).

S.8 FURTHER EXAMPLES OF INCOHERENCY

In Section 5, it has been mentioned that it is possible for the BMA-POCRM to be incoherent without a change of the selected ordering, which can be counter-intuitive. The following example is provided to illustrate such cases.

Consider a 2×2 combination with 2 orderings, both orderings have prior probability $p(1) = p(2) = 0.5$. The skeleton used is $\boldsymbol{\alpha} = (0.10, 0.25, 0.80, 0.85)$, and the TTL is 0.30. Patients are entered one-at-a-time. Suppose after 11 patients, we have

	A		A	
B	a_1	a_2	a_1	a_2
	Number of patients		Number of DLTs	
b_2	2	0	1	0
b_1	0	9	0	5

The estimation results are shown in Table S8 below. After evaluating the first 11 patients, $\tilde{d}_{1,1}$ has estimated toxicity probability closest to the TTL. The 12th and 13th patients are both enrolled to $\tilde{d}_{1,1}$, which results in two non-DLTs. Estimation coherency would require the estimated DLT probabilities of all four combinations to decrease after observing the non-DLT.

However, from the 11th to 12th patient, neither the POCRM nor the BMA-POCRM is coherent. Under both the POCRM and BMA-POCRM, the estimation for d_3 increases from 0.46 to 0.73 under the POCRM and

from 0.581 to 0.584 under the BMA-POCRM. From the 12th to the 13th patient, the POCRM is coherent, the estimated DLT probabilities under all 4 combinations decrease. The BMA-POCRM is incoherent. The DLT probability of d_2 increases from 0.584 to 0.589. Nevertheless, note that the magnitudes of incoherence under the BMA-POCRM, M_A, are 0.003 and 0.005 under the two cases, whereas the magnitude of incoherence under the POCRM, M_S, is 0.273, which is much larger than M_A.

Method	Cohort	$p(m\|\text{Data})$			
		$\mathcal{O}_1$	$\mathcal{O}_2$		
POCRM	11	0.555	0.445		
	12	0.484	0.516		
	13	0.422	0.578		
		$\tilde{a}_i^{(m)}$			
		$\mathcal{O}_1$	$\mathcal{O}_2$		
POCRM	11	0.565	1.369		
	12	0.611	1.411		
	13	0.653	1.449		
		DLT probabilities			
		$\tilde{d}_{1,1}$	$\tilde{d}_{2,1}$	$\tilde{d}_{1,2}$	$\tilde{d}_{2,2}$
POCRM	11	0.272	0.457	0.882	0.912
	12	0.039	0.730	0.141	0.795
	13	0.036	0.724	0.134	0.790
BMA-POCRM	11	0.170	0.581	0.556	0.862
	12	0.138	0.584	0.495	0.848
	13	0.114	0.589	0.442	0.836

Table S8. Estimation results under the POCRM and BMA-POCRM.

S.9 MITIGATING ESTIMATION INCOHERENCIES WITH THE TWO-STAGE POCRM

We conducted a pilot analysis as to whether the two-stage POCRM, which features a run-in phase before applying model selection will reduce the occurrence of estimation incoherencies. The run-in proceeds by enrolling one patient at each dose level in the sequence $d_1 \rightarrow d_2 \rightarrow \ldots \rightarrow d_{16}$ until the first DLT is observed. We carry out our simulation under Scenario 1 of the 4×4 grid scenarios. With the two-stage POCRM, at least one estimation incoherency was present in 74.6% of simulated trials with on average 2.56 estimation incoherencies per trial. This is lower than the standard POCRM, which had at least one estimation incoherency in 84.7% of simulated trials with on average 3.25 incoherencies per trial. We have added these results to Section YY in the Supplementary Materials.

S.10 COMPARISON OF THE RATE OF ONE-SIDED AND TWO-SIDED INCOHERENCIES

In Figures S9 and S10 we show that there is a slight difference in the magnitude of one-sided and two-sided incoherencies, respectively. The magnitudes appear higher in Figure S10 for the two-sided incoherencies. Since this is a more strict criterion than one-sided incoherency, this is expected.

Next, in Figures S11 and S12 we show that $> 99\%$ of estimation incoherencies under all simulation scenarios and under the POCRM model for both one-sided and two-sided incoherencies occur following a change in the selected dose ordering model. Since BMA-POCRM does not select a single dose ordering, this cannot cause incoherencies. The remaining 1% of incoherencies are caused by numerical estimation errors encountered for larger grid sizes, such as the 4×4 grid sizes used in this work. The theoretical results described in Section 5 support this, as they show that under 2×2 grids that estimation incoherencies in the POCRM can only be caused by changes in model selection, and that the BMA-POCRM.

In Figures S13 and S14, it is clear that one-sided and two-sided incoherencies occur at similar rates across scenarios. Additionally, the average number incoherencies under each definition is also similar as shown in Figures S15 and S16.

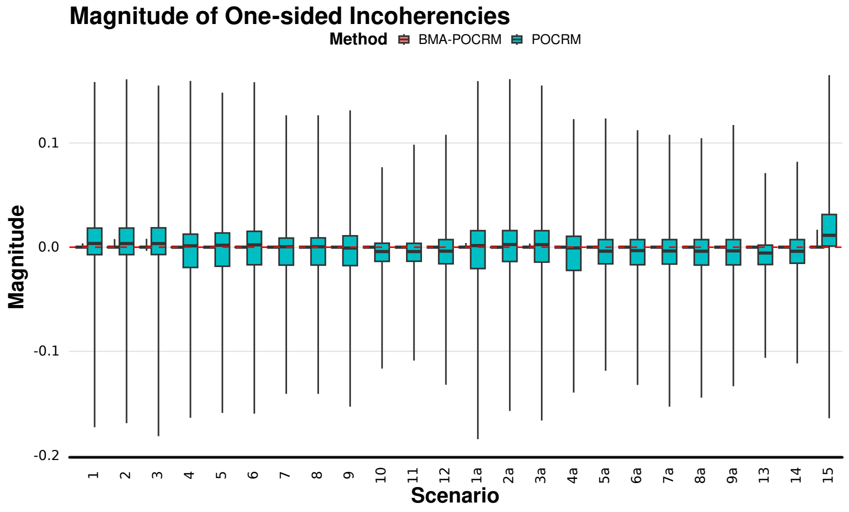

Fig. S9. Distribution of the magnitude of observed one-sided incoherencies under 4×4 grid simulations.

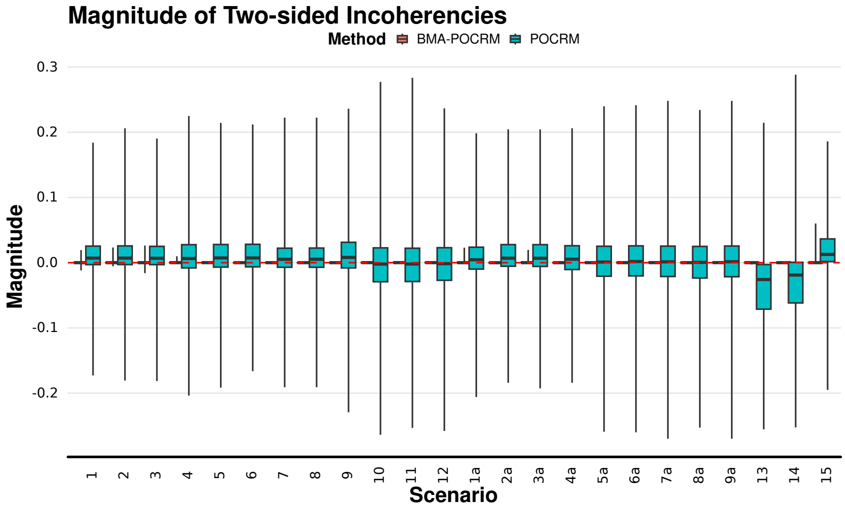

Fig. S10. Distribution of the magnitude of observed two-sided incoherencies under 4×4 grid simulations.

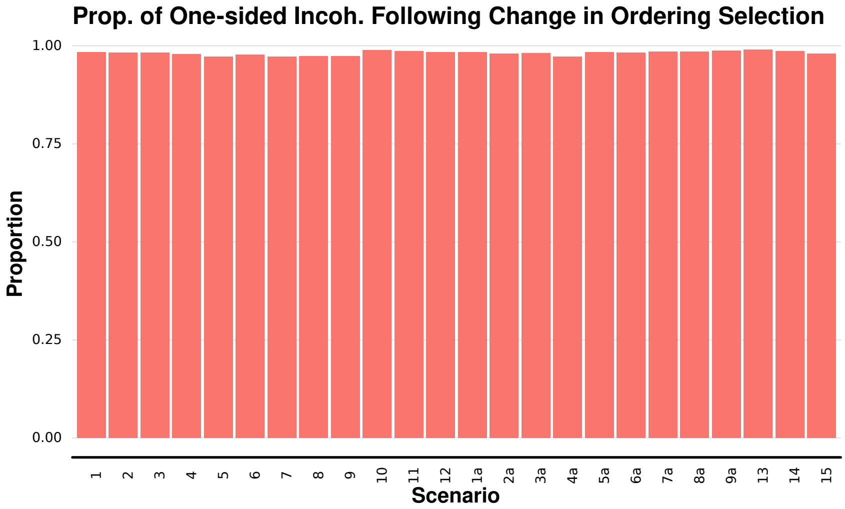

Fig. S11. Proportion of one-sided estimation incoherencies that occur following a change in m^*, the selected ordering under POCRM.

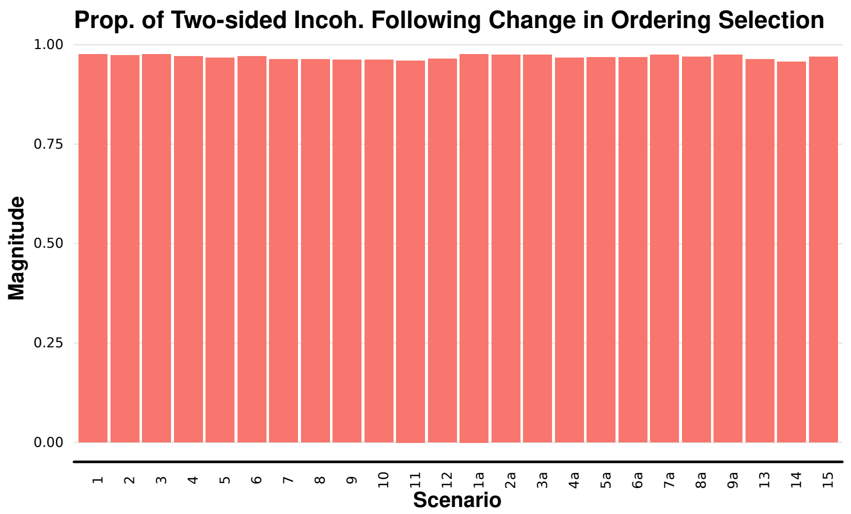

Fig. S12. Proportion of two-sided estimation incoherencies that occur following a change in m^*, the selected ordering under POCRM.

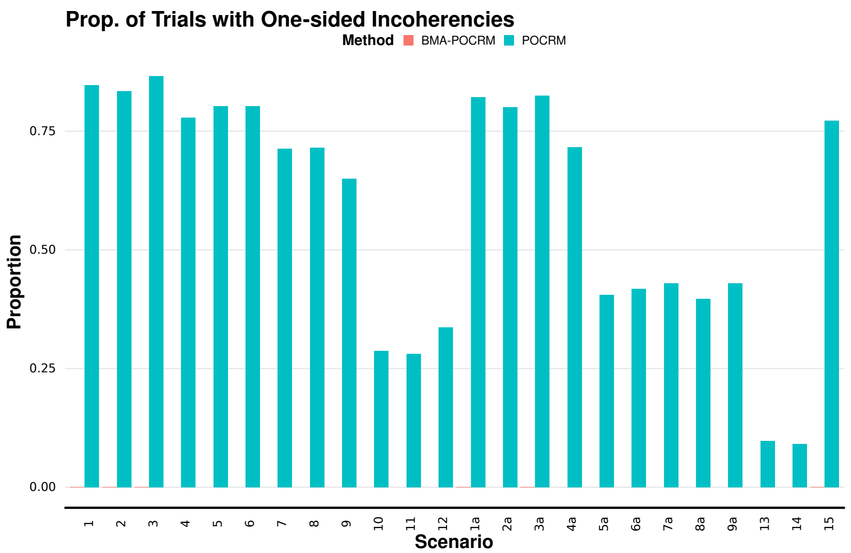

Fig. S13. Proportion of trials with at least one one-sided incoherency.

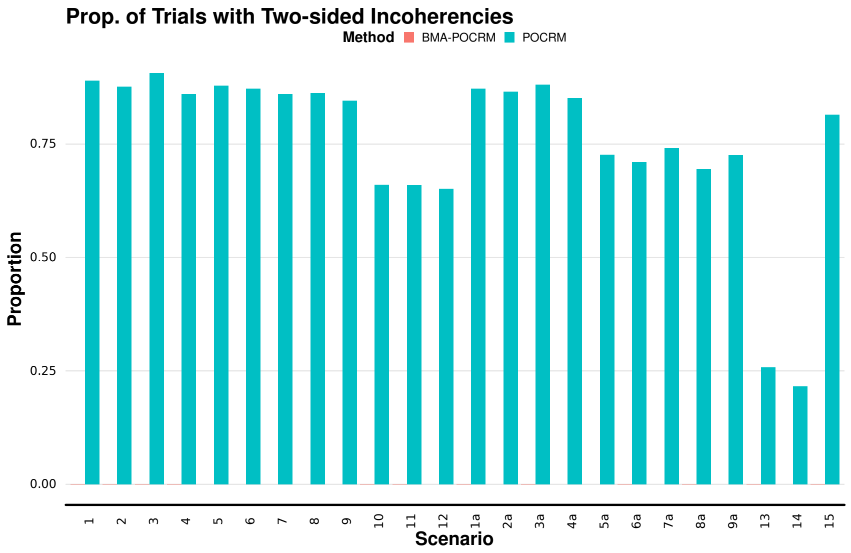

Fig. S14. Proportion of trials with at least one two-sided incoherency.

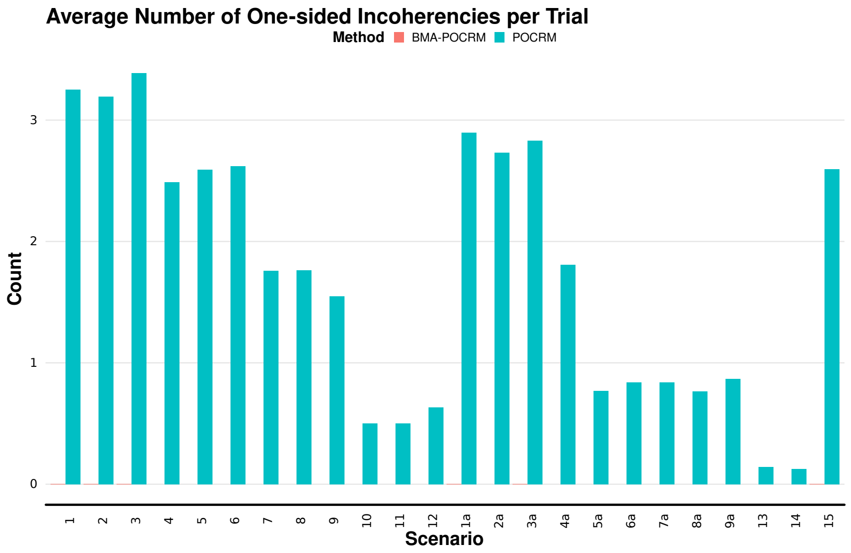

Fig. S15. Average number of one-sided incoherencies per trial.

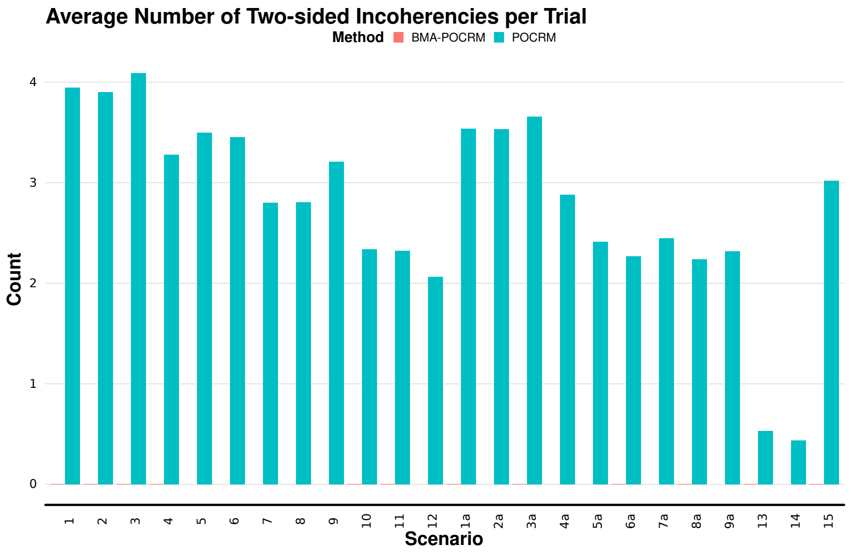

Fig. S16. Average number of two-sided incoherencies per trial.
