# Supplementary material for: Bayesian model averaging for partial ordering continual reassessment methods
Source: Biostatistics. 2025 Oct 21;26(1):kxaf035. doi: 10.1093/biostatistics/kxaf035 (PMC12538209; doi:10.1093/biostatistics/kxaf035)
Supplement: kxaf035_Supplementary_Data [file kxaf035_supplementary_data.zip › biosts-24325-File003.zip!/figures/bma_calib.pdf]

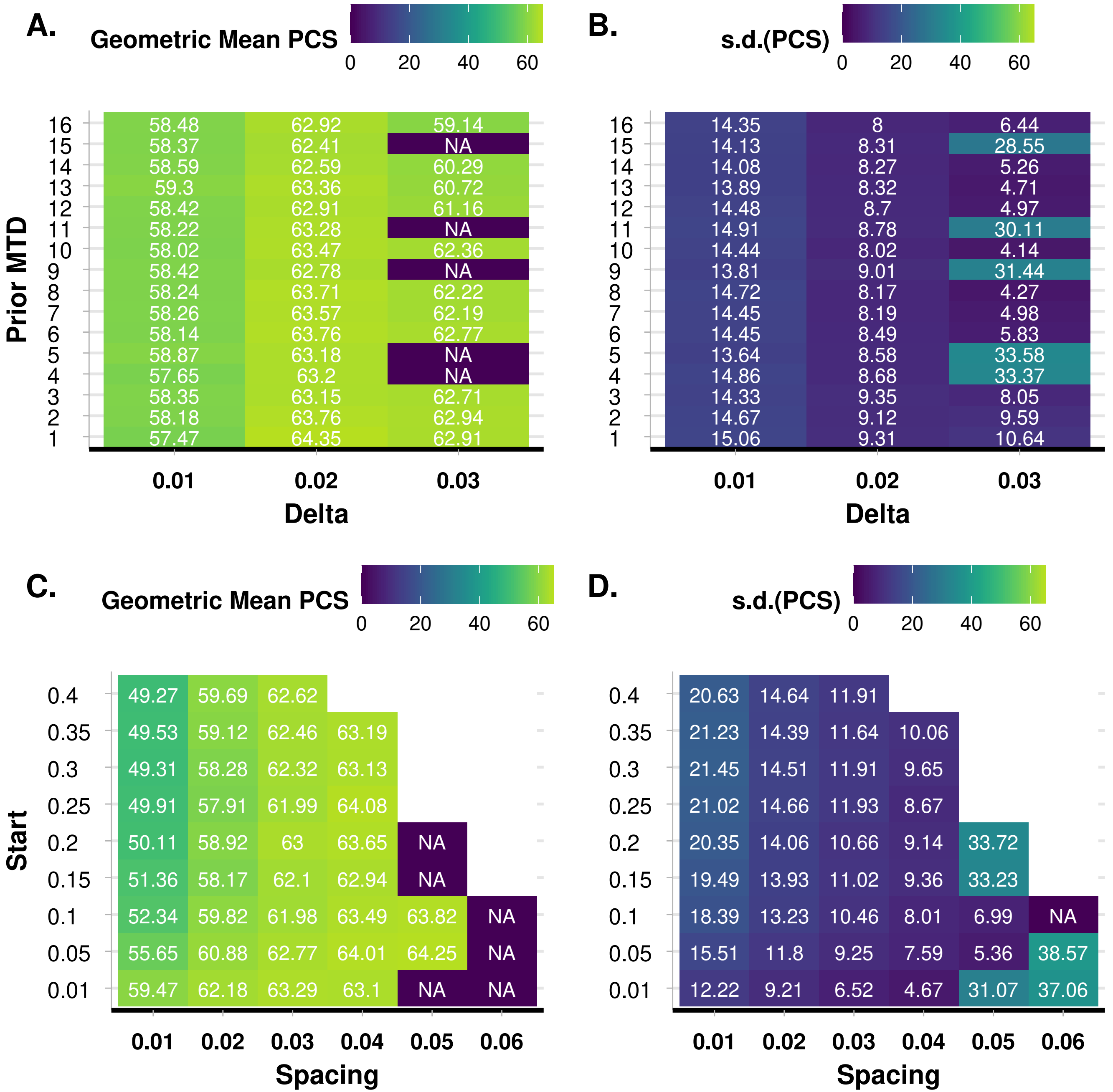
A.
Geometric Mean PCS
0 20 40 60
Prior MTD
16 58.48 62.92 59.14
15 58.37 62.41 NA
14 58.59 62.59 60.29
13 59.3 63.36 60.72
12 58.42 62.91 61.16
11 58.22 63.28 NA
10 58.02 63.47 62.36
9 58.42 62.78 NA
8 58.24 63.71 62.22
7 58.26 63.57 62.19
6 58.14 63.76 62.77
5 58.87 63.18 NA
4 57.65 63.2 NA
3 58.35 63.15 62.71
2 58.18 63.76 62.94
1 57.47 64.35 62.91
0.01 0.02 0.03
Delta
B.
s.d.(PCS)
0 20 40 60
16 14.35 8 6.44
15 14.13 8.31 28.55
14 14.08 8.27 5.26
13 13.89 8.32 4.71
12 14.48 8.7 4.97
11 14.91 8.78 30.11
10 14.44 8.02 4.14
9 13.81 9.01 31.44
8 14.72 8.17 4.27
7 14.45 8.19 4.98
6 14.45 8.49 5.83
5 13.64 8.58 33.58
4 14.86 8.68 33.37
3 14.33 9.35 8.05
2 14.67 9.12 9.59
1 15.06 9.31 10.64
0.01 0.02 0.03
Delta
C.
Geometric Mean PCS
0 20 40 60
Start
0.4 49.27 59.69 62.62
0.35 49.53 59.12 62.46 63.19
0.3 49.31 58.28 62.32 63.13
0.25 49.91 57.91 61.99 64.08
0.2 50.11 58.92 63 63.65 NA
0.15 51.36 58.17 62.1 62.94 NA
0.1 52.34 59.82 61.98 63.49 63.82 NA
0.05 55.65 60.88 62.77 64.01 64.25 NA
0.01 59.47 62.18 63.29 63.1 NA NA
0.01 0.02 0.03 0.04 0.05 0.06
Spacing
D.
s.d.(PCS)
0 20 40 60
0.4 20.63 14.64 11.91
0.35 21.23 14.39 11.64 10.06
0.3 21.45 14.51 11.91 9.65
0.25 21.02 14.66 11.93 8.67
0.2 20.35 14.06 10.66 9.14 33.72
0.15 19.49 13.93 11.02 9.36 33.23
0.1 18.39 13.23 10.46 8.01 6.99 NA
0.05 15.51 11.8 9.25 7.59 5.36 38.57
0.01 12.22 9.21 6.52 4.67 31.07 37.06
0.01 0.02 0.03 0.04 0.05 0.06
Spacing
