# Supplementary material for: Bayesian model averaging for partial ordering continual reassessment methods
Source: Biostatistics. 2025 Oct 21;26(1):kxaf035. doi: 10.1093/biostatistics/kxaf035 (PMC12538209; doi:10.1093/biostatistics/kxaf035)
Supplement: kxaf035_Supplementary_Data [file kxaf035_supplementary_data.zip › biosts-24325-File003.zip!/figures/coh36_plot.pdf]

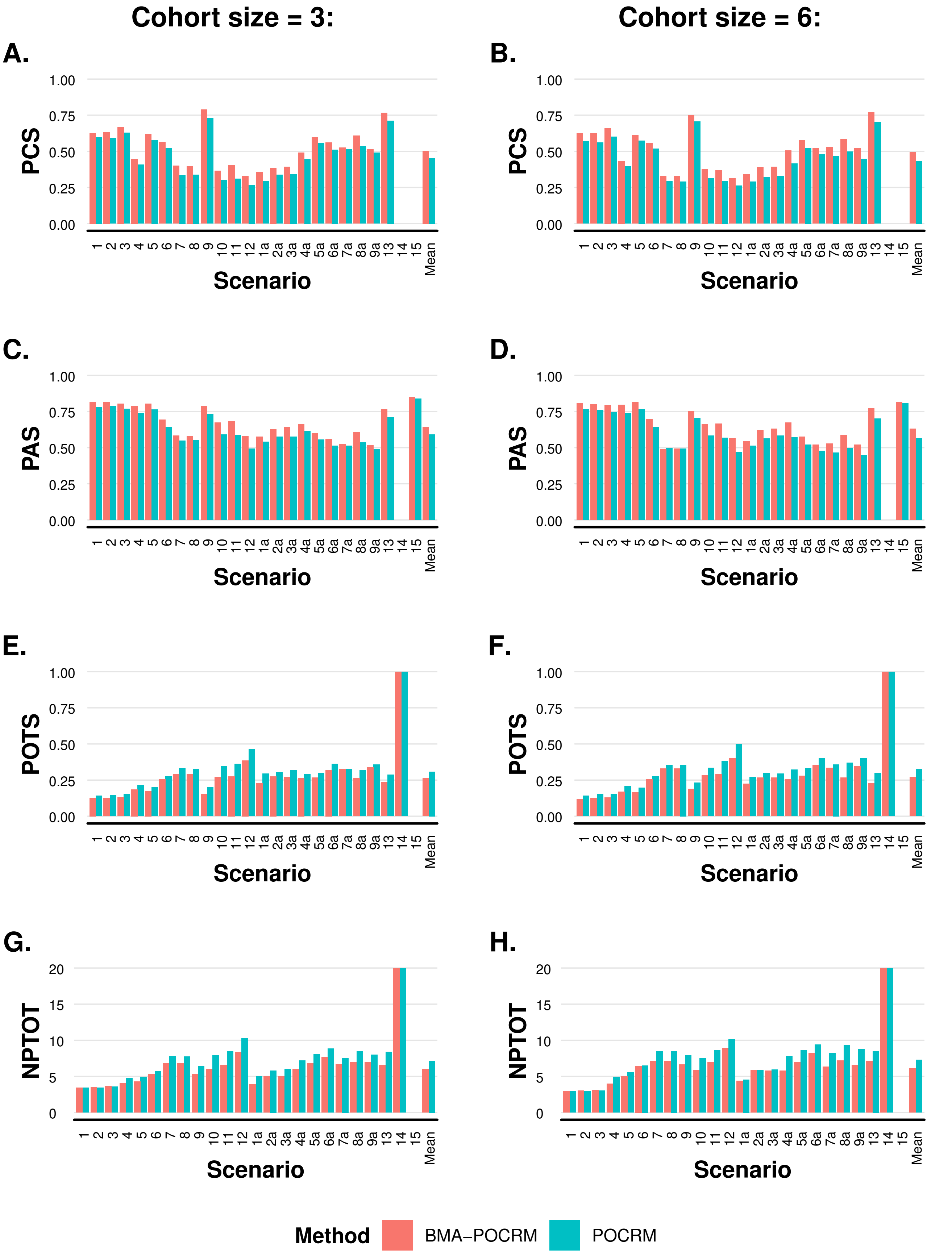

Cohort size = 3:
Cohort size = 6:
A.
B.
C.
D.
E.
F.
G.
H.
PCS
PAS
POTS
NPTOT
Scenario
1 2 3 4 5 6 7 8 9 10 11 12 1a 2a 3a 4a 5a 6a 7a 8a 9a 13 14 15 Mean
Method
BMA−POCRM
POCRM
