# Supplementary material for: Bayesian model averaging for partial ordering continual reassessment methods
Source: Biostatistics. 2025 Oct 21;26(1):kxaf035. doi: 10.1093/biostatistics/kxaf035 (PMC12538209; doi:10.1093/biostatistics/kxaf035)
Supplement: kxaf035_Supplementary_Data [file kxaf035_supplementary_data.zip › biosts-24325-File003.zip!/figures/combined_plot_2x3grid.pdf]

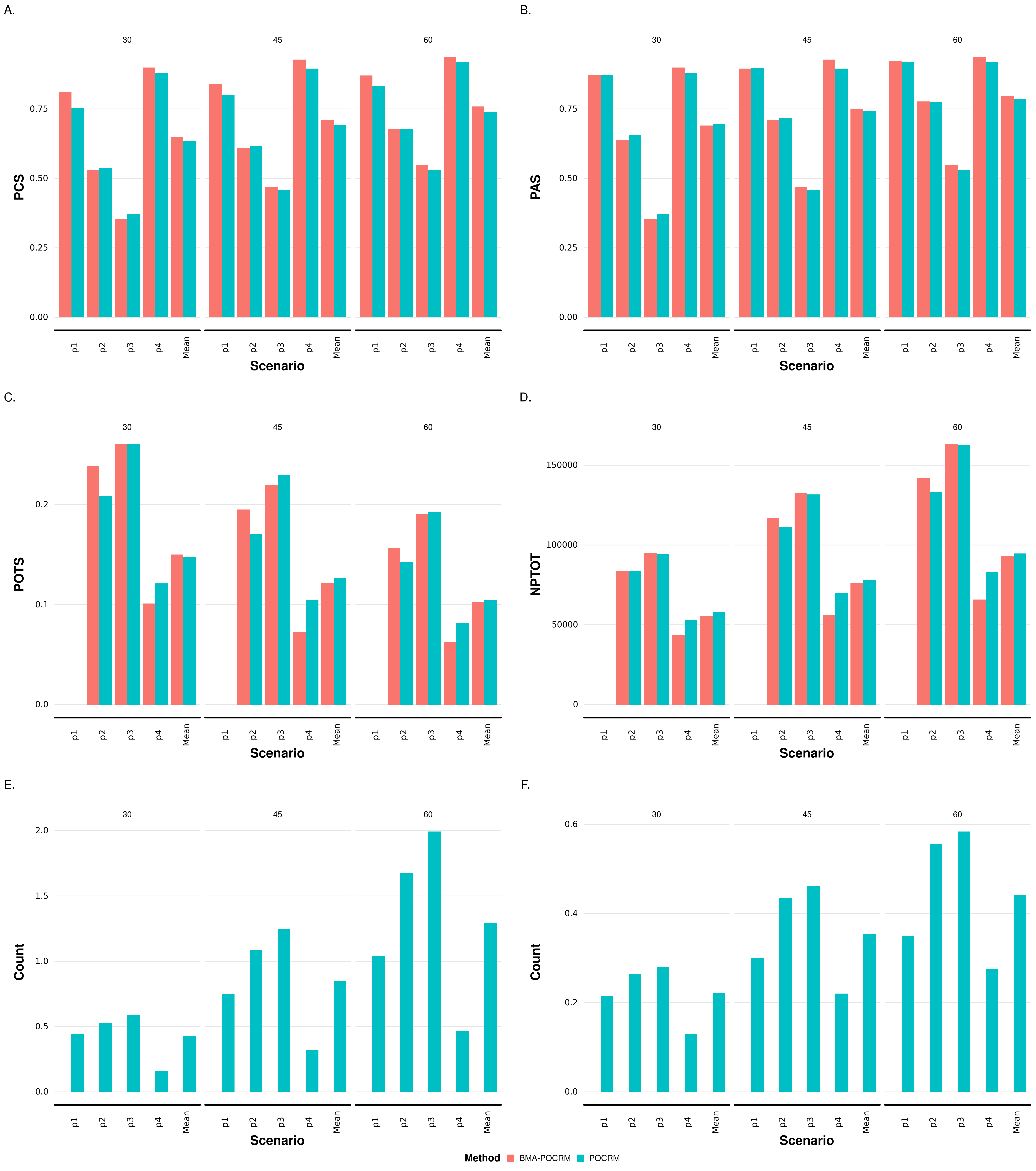

A.
B.
C.
D.
E.
F.
30
45
60
PCS
PAS
POTS
NPTOT
Count
Scenario
p1
p2
p3
p4
Mean
Method
BMA-POCRM
POCRM
