# Supplementary material for: Bayesian model averaging for partial ordering continual reassessment methods
Source: Biostatistics. 2025 Oct 21;26(1):kxaf035. doi: 10.1093/biostatistics/kxaf035 (PMC12538209; doi:10.1093/biostatistics/kxaf035)
Supplement: kxaf035_Supplementary_Data [file kxaf035_supplementary_data.zip › biosts-24325-File003.zip!/figures/incoh_plot_final.pdf]

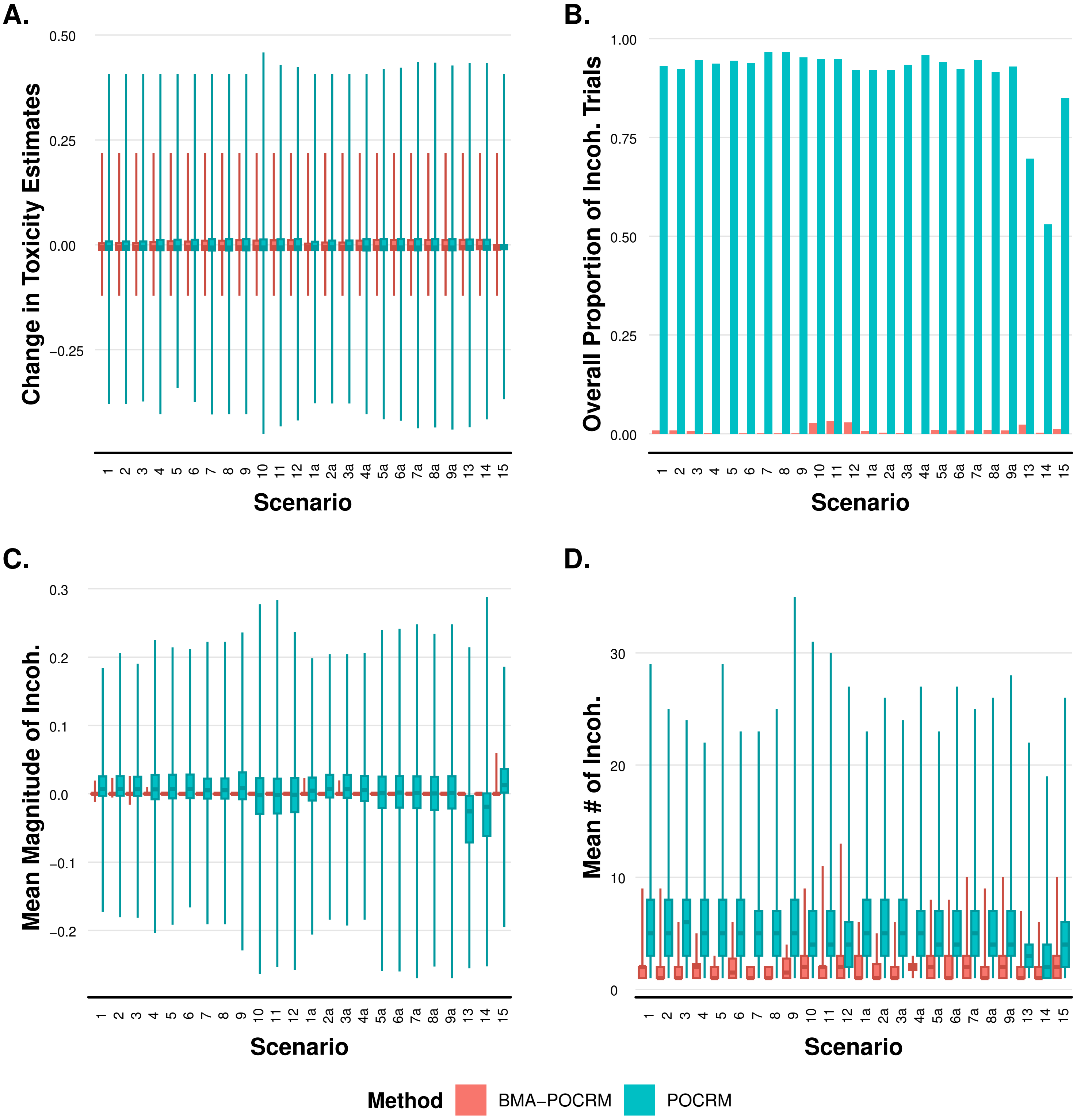
A.
Change in Toxicity Estimates
0.50
0.25
0.00
−0.25
Scenario
B.
Overall Proportion of Incoh. Trials
1.00
0.75
0.50
0.25
0.00
Scenario
C.
Mean Magnitude of Incoh.
0.3
0.2
0.1
0.0
−0.1
−0.2
Scenario
D.
Mean # of Incoh.
30
20
10
0
Scenario
1 2 3 4 5 6 7 8 9 10 11 12 1a 2a 3a 4a 5a 6a 7a 8a 9a 13 14 15
Method
BMA−POCRM
POCRM
