# Supplementary material for: Bayesian model averaging for partial ordering continual reassessment methods
Source: Biostatistics. 2025 Oct 21;26(1):kxaf035. doi: 10.1093/biostatistics/kxaf035 (PMC12538209; doi:10.1093/biostatistics/kxaf035)
Supplement: kxaf035_Supplementary_Data [file kxaf035_supplementary_data.zip › biosts-24325-File003.zip!/figures/orderchange_m.pdf]

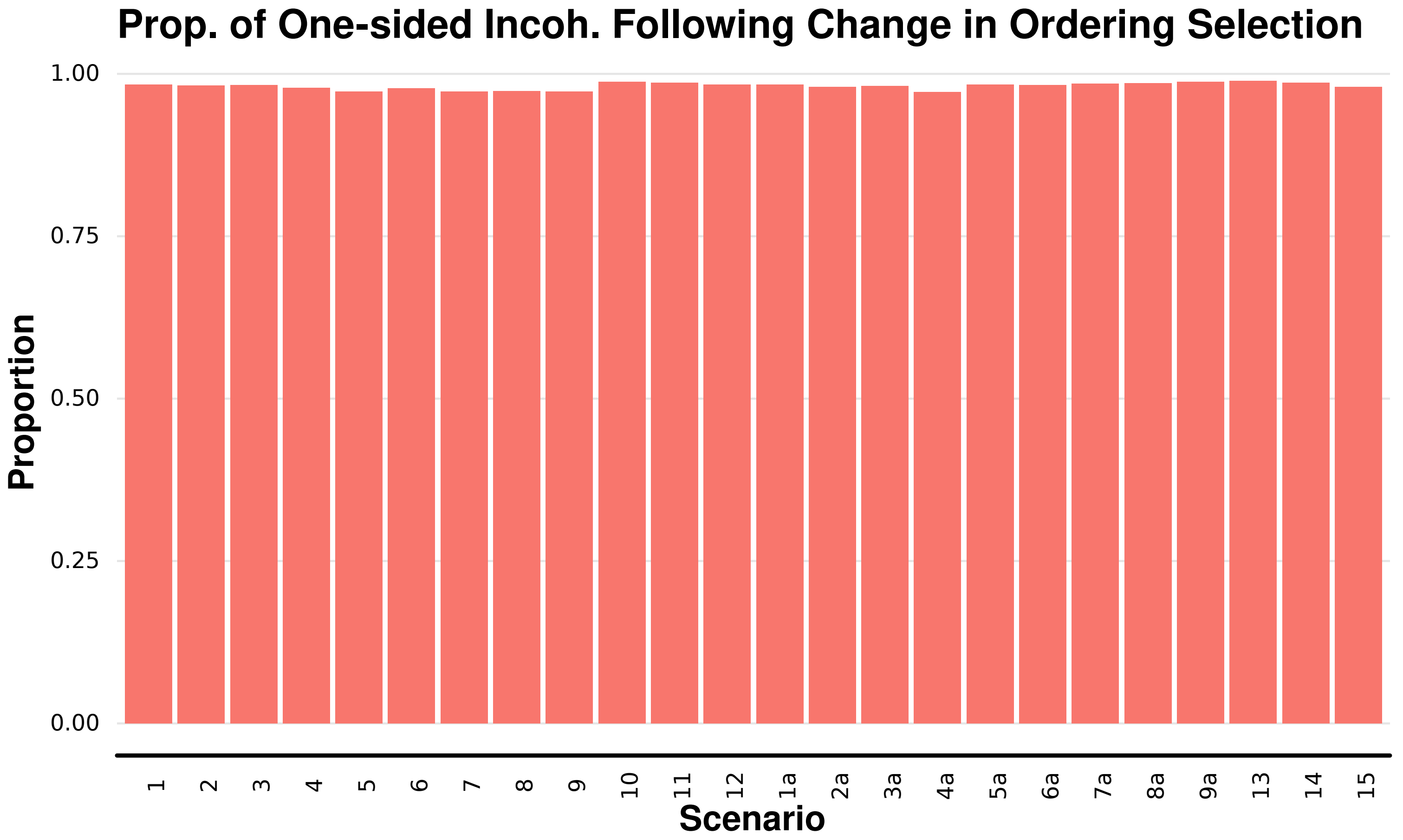

Prop. of One-sided Incoh. Following Change in Ordering Selection
Proportion
1.00
0.75
0.50
0.25
0.00
1
2
3
4
5
6
7
8
9
10
11
12
1a
2a
3a
4a
5a
6a
7a
8a
9a
13
14
15
Scenario
