# Supplementary material for: Bayesian model averaging for partial ordering continual reassessment methods
Source: Biostatistics. 2025 Oct 21;26(1):kxaf035. doi: 10.1093/biostatistics/kxaf035 (PMC12538209; doi:10.1093/biostatistics/kxaf035)
Supplement: kxaf035_Supplementary_Data [file kxaf035_supplementary_data.zip › biosts-24325-File003.zip!/figures/plot_mag_onesided.pdf]

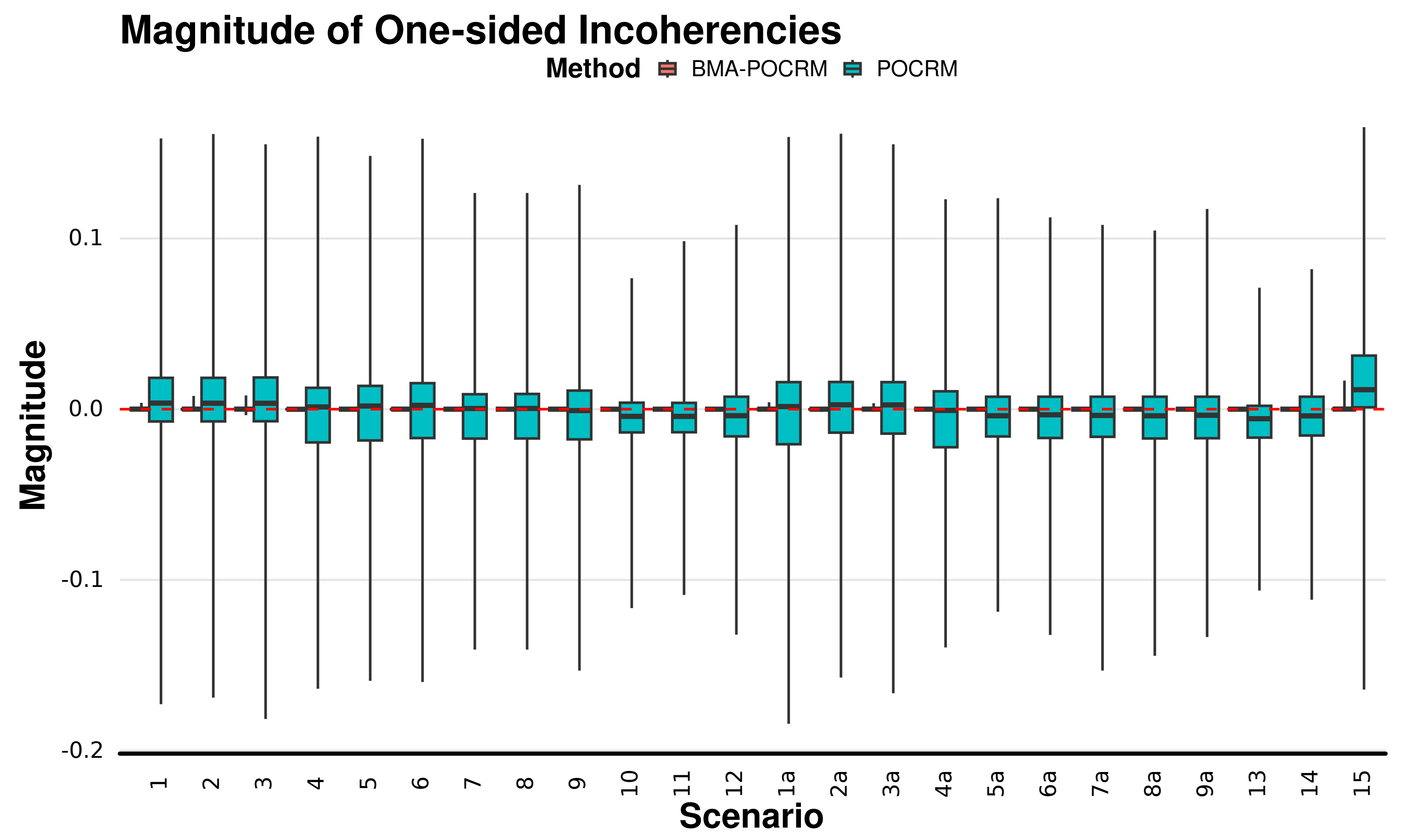
Magnitude of One-sided Incoherencies
Method
BMA-POCRM
POCRM
0.1
0.0
-0.1
-0.2
Magnitude
1
2
3
4
5
6
7
8
9
10
11
12
1a
2a
3a
4a
5a
6a
7a
8a
9a
13
14
15
Scenario
