# Supplementary material for: Bayesian model averaging for partial ordering continual reassessment methods
Source: Biostatistics. 2025 Oct 21;26(1):kxaf035. doi: 10.1093/biostatistics/kxaf035 (PMC12538209; doi:10.1093/biostatistics/kxaf035)
Supplement: kxaf035_Supplementary_Data [file kxaf035_supplementary_data.zip › biosts-24325-File003.zip!/figures/real_trial_jump_final.pdf]

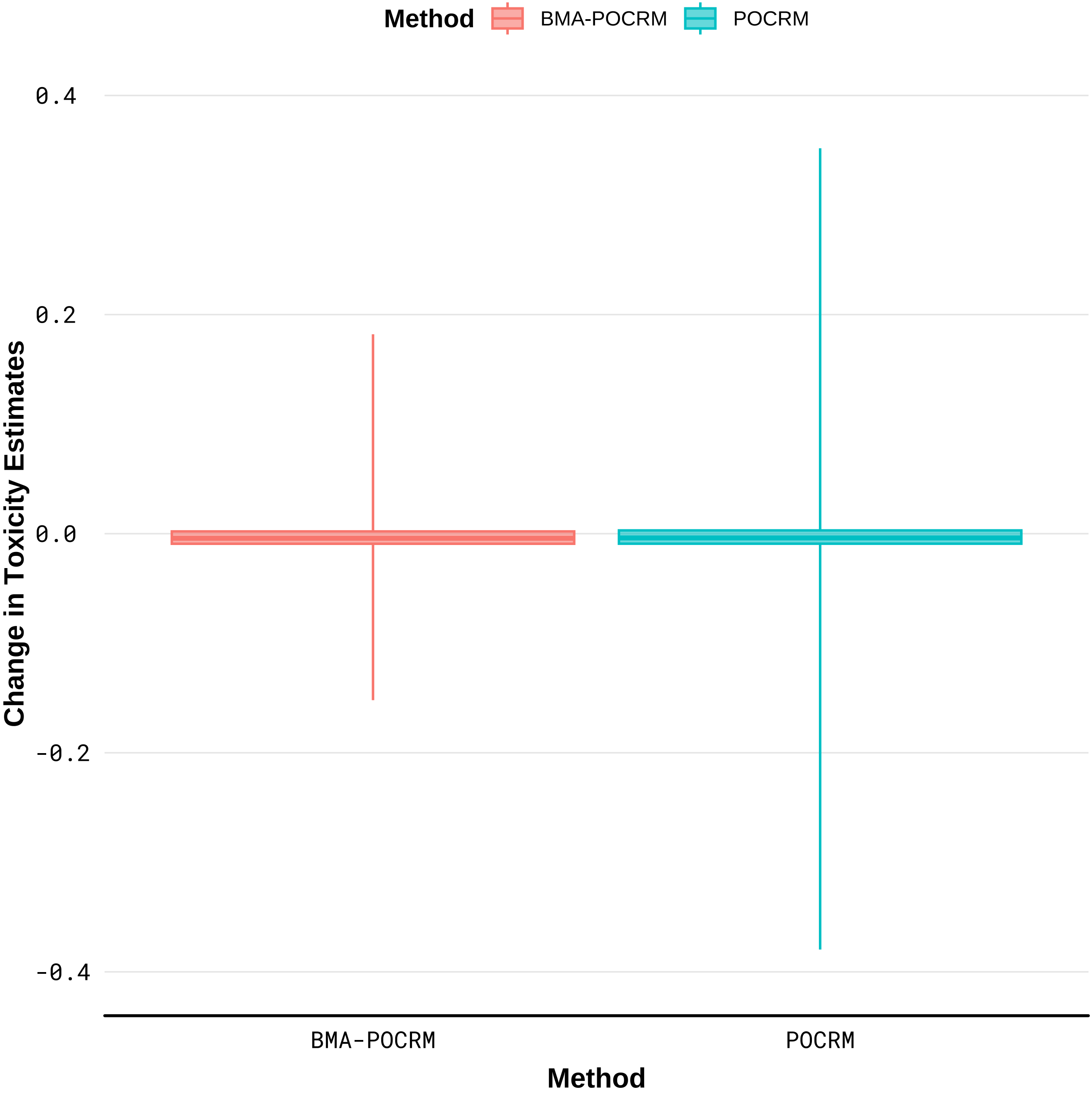

Method
BMA-POCRM
POCRM
0.4
0.2
0.0
-0.2
-0.4
Change in Toxicity Estimates
BMA-POCRM
POCRM
Method
