# Supplementary material for: Bayesian model averaging for partial ordering continual reassessment methods
Source: Biostatistics. 2025 Oct 21;26(1):kxaf035. doi: 10.1093/biostatistics/kxaf035 (PMC12538209; doi:10.1093/biostatistics/kxaf035)
Supplement: kxaf035_Supplementary_Data [file kxaf035_supplementary_data.zip › biosts-24325-File003.zip!/figures/trial_incoherencies.pdf]

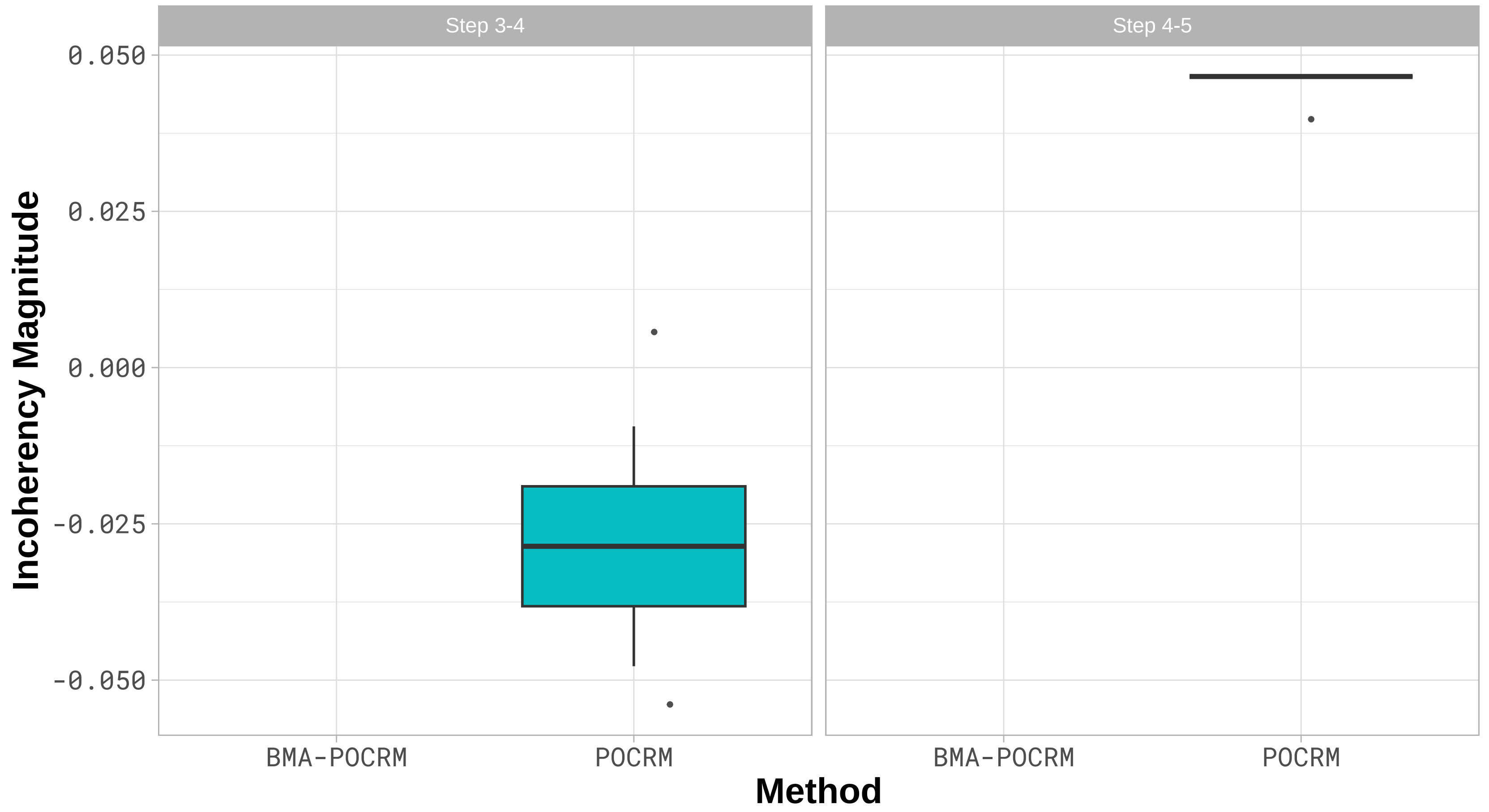

Step 3-4
Step 4-5
0.050
0.025
0.000
-0.025
-0.050
Incoherency Magnitude
BMA-POCRM
POCRM
BMA-POCRM
POCRM
Method
