# Supplementary material for: Bayesian model averaging for partial ordering continual reassessment methods
Source: Biostatistics. 2025 Oct 21;26(1):kxaf035. doi: 10.1093/biostatistics/kxaf035 (PMC12538209; doi:10.1093/biostatistics/kxaf035)
Supplement: kxaf035_Supplementary_Data [file kxaf035_supplementary_data.zip › biosts-24325-File003.zip!/figures/vm_var_plot.pdf]

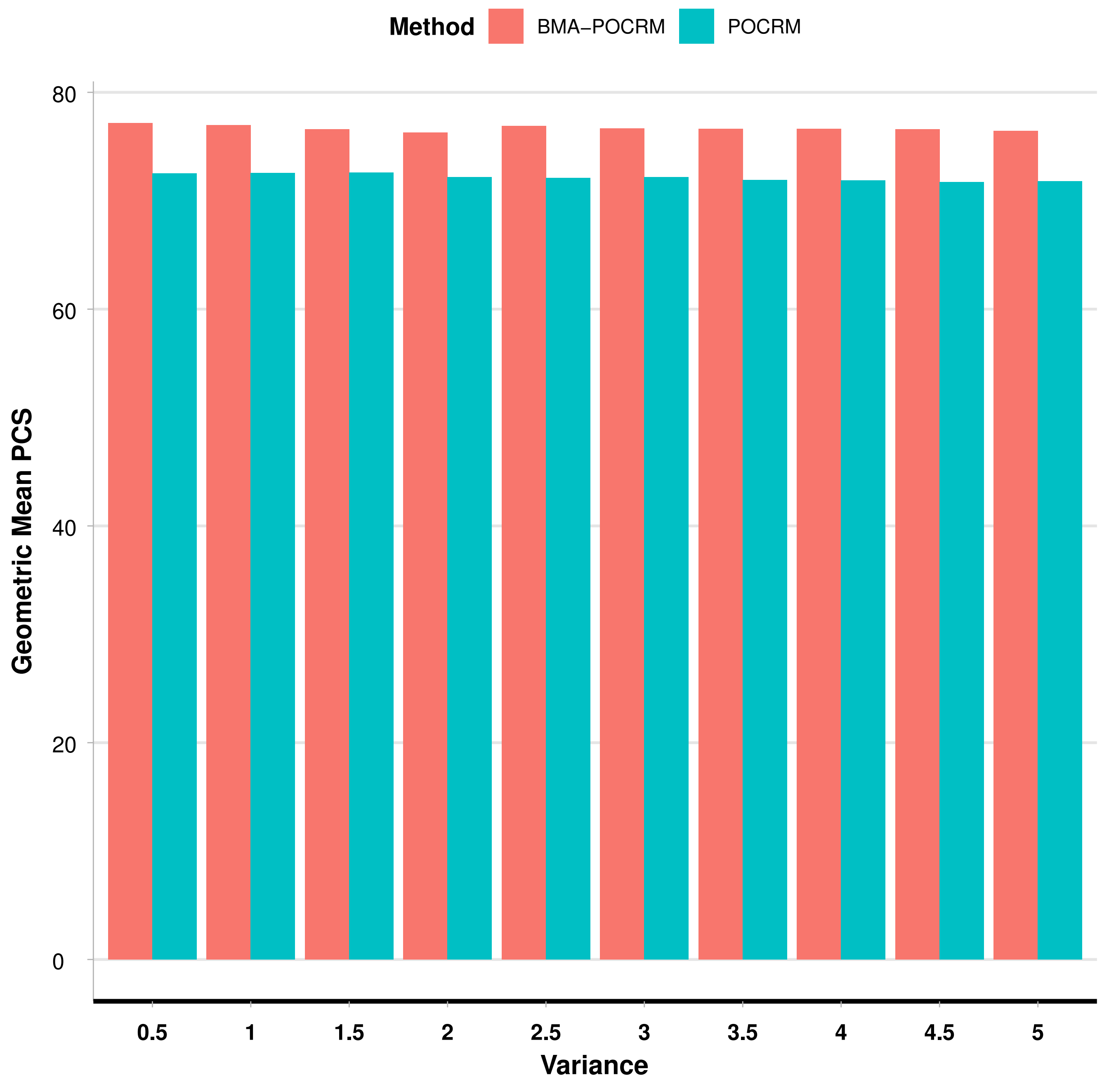

Method
BMA−POCRM
POCRM
Geometric Mean PCS
0
20
40
60
80
0.5
1
1.5
2
2.5
3
3.5
4
4.5
5
Variance
